# Reproducible generation of human retinal ganglion cells from banked retinal progenitor cells: analysis of target recognition and IGF-1-mediated axon regeneration

**DOI:** 10.3389/fcell.2023.1214104

**Published:** 2023-07-13

**Authors:** Murali Subramani, Matthew J. Van Hook, Iqbal Ahmad

**Affiliations:** Department of Ophthalmology and Visual Science, University of Nebraska Medical Center, Omaha, NE, United States

**Keywords:** glaucoma, RGC, human, regeneration, IGF-1, mTOR, disease-modeling, transplantation

## Abstract

The selective degeneration of retinal ganglion cells (RGCs) is a common feature in glaucoma, a complex group of diseases, leading to irreversible vision loss. Stem cell-based glaucoma disease modeling, cell replacement, and axon regeneration are viable approaches to understand mechanisms underlying glaucomatous degeneration for neuroprotection, *ex vivo* stem cell therapy, and therapeutic regeneration. These approaches require direct and facile generation of human RGCs (hRGCs) from pluripotent stem cells. Here, we demonstrate a method for rapid generation of hRGCs from banked human pluripotent stem cell-derived retinal progenitor cells (hRPCs) by recapitulating the developmental mechanism. The resulting hRGCs are stable, functional, and transplantable and have the potential for target recognition, demonstrating their suitability for both *ex vivo* stem cell approaches to glaucomatous degeneration and disease modeling. Additionally, we demonstrate that hRGCs derived from banked hRPCs are capable of regenerating their axons through an evolutionarily conserved mechanism involving insulin-like growth factor 1 and the mTOR axis, demonstrating their potential to identify and characterize the underlying mechanism(s) that can be targeted for therapeutic regeneration.

## Introduction

The retina is an integral part of the central nervous system (CNS), consisting of seven different cell types organized in a stereotypical laminar organization. These cells are generated from multipotential retinal progenitor cells (RPCs) in an evolutionarily conserved temporal sequence in response to cell–cell interactions, recruiting cell-type-specific transcription factors (Ahmad et al., 2020). The retinal ganglion cells (RGCs), which are born first (Young, 1985; Rapaport et al., 2004), are the main output neurons relaying the visual signal generated by the photoreceptors to the higher centers in the brain for the perception of vision. The degeneration of RGCs in glaucoma, a complex and multifactorial disease, leads to irreversible blindness (Almasieh et al., 2012). Unfortunately, there is no effective treatment to reverse the loss of vision when RGCs die. However, research over the last decade has led to discoveries that are promising for regenerative medicine for glaucomatous RGC degeneration. These include the directed differentiation of pluripotent stem cells, embryonic stem cells (ESCs) or induced pluripotent stem cells (iPSCs), in 2D culture into RGCs (Parameswaran et al., 2015; Ohlemacher et al., 2016; Teotia et al., 2017a; Sluch et al., 2017; Ahmad et al., 2020) and self-organization of pluripotent stem cells into 3D retinal organoids (Eiraku et al., 2011; Nakano et al., 2012; Kuwahara et al., 2015; Volkner et al., 2016; Langer et al., 2018), providing platforms for disease modeling of glaucoma (Tucker et al., 2013; Ohlemacher et al., 2016; Teotia et al., 2017c; Ahmad et al., 2020; VanderWall et al., 2020) and cells for retinal repair (Venugopalan et al., 2016; Oswald et al., 2021; Zhang et al., 2021). Although retinal organoids have a clear advantage of generating retinal cells simulating cell–cell interactions of the retinal niche and, hence, reflect conditions closer to a developing retina, the directed differentiation is a facile approach to generate pure populations of functional hRGCs in a relatively short time for *ex vivo* stem cell approaches and/or disease modeling (Ahmad et al., 2020).

The previous method developed in our lab for the directed generation of hRGCs was based on recapitulating the developmental mechanism. It was divided into three phases: initiation, differentiation, and maturation, where stage-specific recruitment of developmentally relevant signaling through small molecules/recombinant growth factors led to the generation of hRGCs with a stable phenotype (Teotia et al., 2017a; Ahmad et al., 2020). The *ex vivo*-generated hRGCs shared a transcription profile with native RGCs (Teotia et al., 2017b) and displayed the electrophysiological signature of RGCs and subtype diversity (Teotia et al., 2017a; Teotia et al., 2017c; Teotia et al., 2019). These cells displayed potential to discriminate between specific and non-specific central targets (Teotia et al., 2017a), integrate in the host retina following transplantation (Ahmad et al., 2020), and support glaucoma disease (Teotia et al., 2017c) and axon regeneration (Teotia et al., 2019) modeling. Here, we demonstrate streamlining of the method that reduces the time for reproducible generation of hRGCs from pluripotent cells to a month through the modification of neural/retinal induction of pluripotent cells in a monolayer culture paradigm. This modification allowed the banking of hRPCs, which reduced the hRGC generation time to half while offering flexibility of their use. In addition to demonstrating the efficiency and fidelity of hRGCs generated from the banked RPCs, our results using these cells show their evolutionarily conserved ability to recognize central retinal targets and IGF-1-mediated axonal regeneration. Hence, the banked RPCs represent a facile and reliable resource for modeling disease and therapeutic regeneration besides supporting an *ex vivo* stem cell approach to glaucomatous degeneration.

## Results

**FIGURE 1:**
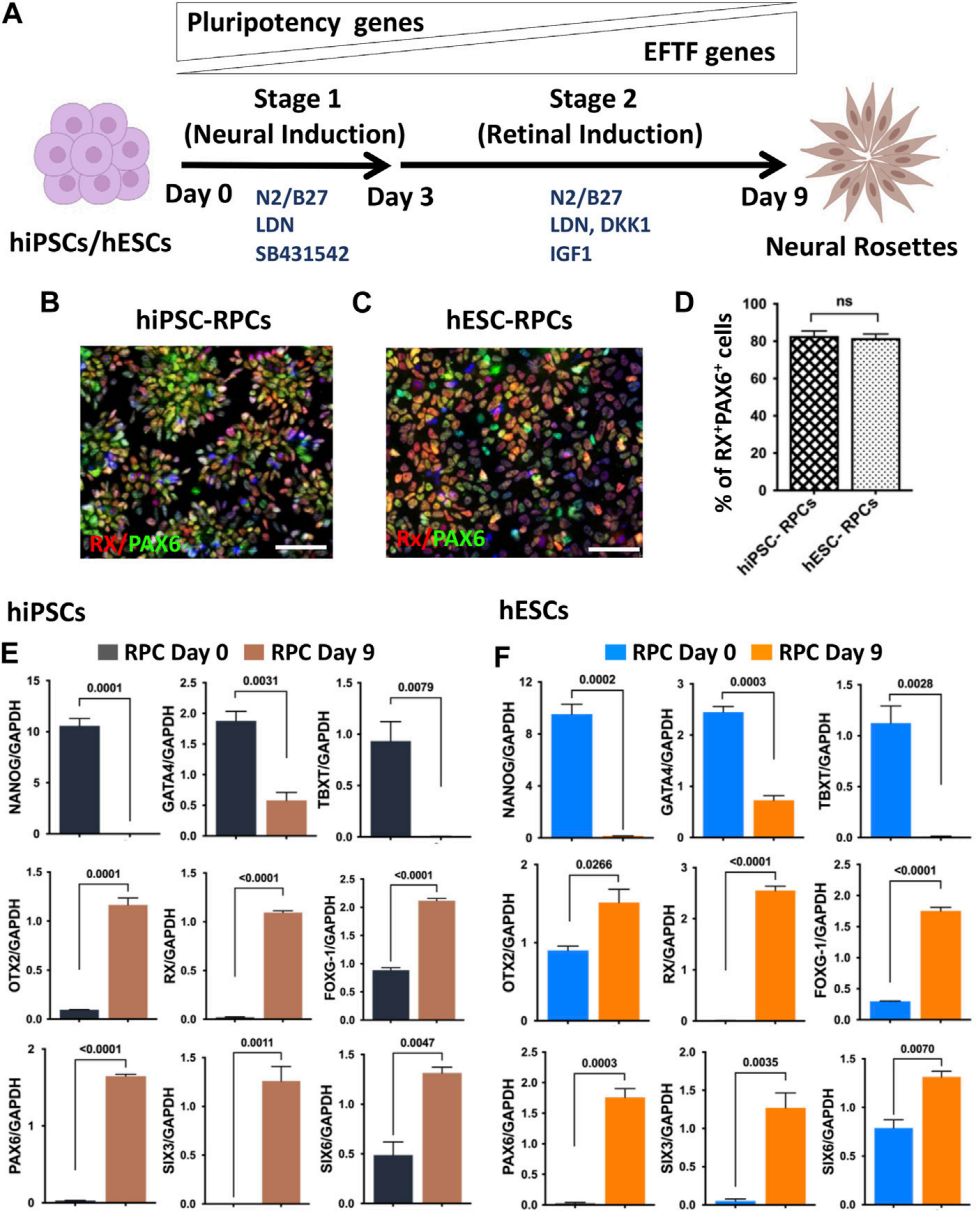
Neural/retinal induction of human pluripotent stem cells. Human pluripotent stem cells (hiPSCs and hESCs) are shown in a schematic representation of neural induction (stage 1) and retinal induction (stage 2), respectively **(A)**. Immunocytochemical analysis of NRs, derived from hiPSCs **(B)** and hESCs **(C)**, at the 9th day of the neural/retinal induction reveals cells co-expressing RPC markers, RX and PAX6 **(D)**. During neural/retinal induction of hiPSCs **(E)** and hESCs **(F)**, there was a temporal silencing of pluripotency (*NANOG*)- and germ layer (*GATA and, TBXT*)-specific genes and reciprocal temporal induction of neuroectodermal (*OTX2*), forebrain (*FOXG1*), and EFTF specific genes (*RX, PAX6, SIX3 and SIX6*), demonstrating the recruitment of the developmental mechanism for differentiation along the retinal lineage. Experiments were carried out in triplicates per group. Scale bars: 50 μm.

**hRPC generation in a monolayer culture paradigm:** The previous method of neural/retinal induction was time consuming and had variations in the efficiency of generation of RPCs depending on the skill of the selection of embryoid bodies (EBs)/neural rosettes (NRs) (Ahmad et al., 2020). We addressed these two drawbacks through retinal induction in the monolayer culture of pluripotent cells. This was achieved in two stages: the first stage involved neural induction using the dual SMAD inhibition protocol (Chambers et al., 2009), and in the second stage, neural progenitors were differentiated along the retinal lineage through the inhibition and activation of Wnt and IGF-1 signaling, respectively (Lamba et al., 2006; Parameswaran et al., 2015; Teotia et al., 2017a) (Figure 1A). We carried out a side-by-side comparison of the efficiency of RPC generation from hiPSCs (Teotia et al., 2017a) and gene-edited hESCs, in which tdT expression is driven by the promoter of the RGC-specific gene, *POU4F2* (Sluch et al., 2017). For pluripotent cells in the monolayer culture to survive, they were plated at a high density (2 × 10^6^ cells/10 cm^2^) on Matrigel-coated plates in a neural induction medium (NIM) containing ROCK inhibitor, Y27632 (Watanabe et al., 2007). Media were changed daily, given the high density of pluripotent cells. In the neural induction stage, pluripotent cells were exposed to two inhibitors of SMAD signaling, Noggin (Elkabetz et al., 2008) and the small molecule, SB431542 (Smith et al., 2008), for first 3 days (0–2 days) to nudge the cells preferentially along the neuroectodermal lineage. In the final protocol, Noggin was replaced by LDN-193189, a selective inhibitor of BMP signaling (Yu et al., 2008). The next 6 days (3–9 days), cells were exposed to DKK1 and IGF-1 along with LDN but without SB431542 to promote differentiation along the retinal lineage. The immunocytochemical analysis at the end of retinal induction revealed that cells co-expressed RX and PAX6 immunoreactivities in NRs and their proportion in the culture, derived from hiPSCs or hESCs^POU4F2−tdT^, was more than 80% (82.83 ± 2.265 hiPSCs *vs*. 81.67 ± 2.556 hESCs) of total cells (Figures 1B–D). To know whether or not the hRPCs generation involved a developmental mechanism, we examined the temporal expression pattern of developmentally regulated genes during the neural/retinal induction. We observed a temporal silencing of the expression of transcripts corresponding to the pluripotency gene (*NANOG*), mesoderm-specific gene (*TBXT*), and endoderm-specific gene *(GATA4*), while those of neuroectoderm (*OTX2*)- and forebrain (*FOXG1*)-specific genes increased by time, suggesting an abrogation of the pluripotency potential and selective induction of both hiPSCs (Figure 1E) and hESCs (Figure 1F) along the neural lineage. There was a temporal increase in transcripts corresponding to eye field transcription factor (EFTF) genes *RX, PAX6, SIX3, and SIX6* (Chow and Lang, 2001), demonstrating subsequent differentiation of neural cells along the retinal lineage (Figures 1E, F). These observations demonstrated that the retinal induction was regulated and not random, therefore predicting their stable phenotype. hRPCs, derived from either hiPSCs or hESCs, were immediately processed (fresh hRPCs) for RGC differentiation or frozen (banked hRPCs) in the NR freezing medium (STEMCELL Technologies) at the density of 5 × 10^6^ cells/ml and stored in liquid N_2_.

**FIGURE 2:**
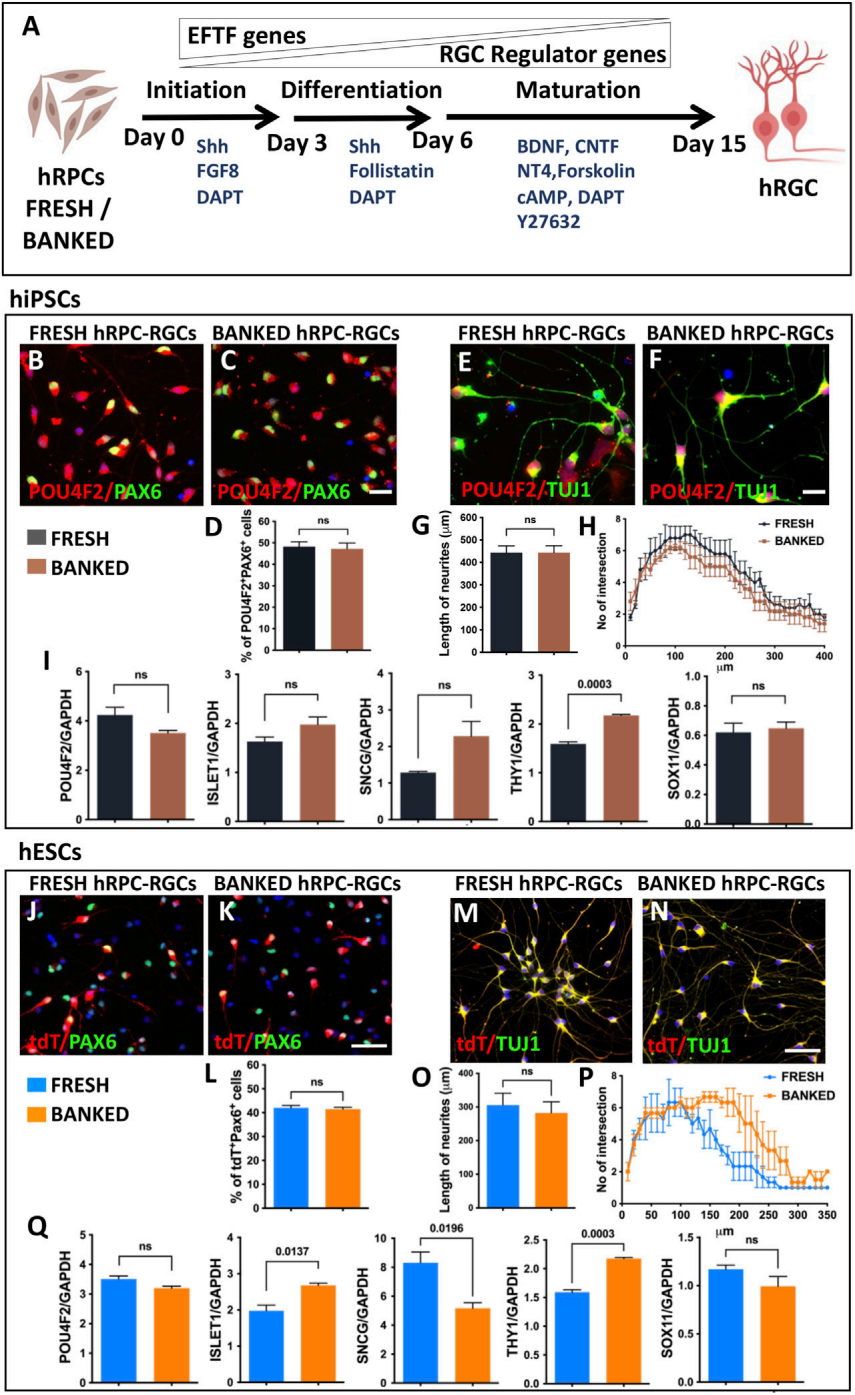
Differentiation of fresh/banked hRPCs derived from human pluripotent stem cells into RGCs. Schematic representation of fresh or banked hRPCs’ differentiation into RGCs by stage-specific recruitment of the developmental mechanism **(A)**. Immunocytochemical analysis of RGCs, differentiated from hiPSC-derived fresh **(B)** and banked **(C)** RPCs, at the 16th day of RGC co-expressed immunoreactivities corresponding to RGC regulators, POU4F2 and PAX6. The proportion of cells co-expressing POU4F2 and PAX6 immunoreactivities in the culture is given in the graph **(D)**. Immunocytochemical analysis of neurites of RGCs, differentiated from hiPSC-derived fresh **(E)** and banked **(F)** RPCs, reveals the relative length **(G)** and their complexity **(H)** by Sholl analysis. qPCR analysis of RGCs, derived from fresh and banked hRPCs, reveals the relative expression of transcripts corresponding to RGC regulators and marker genes **(I)**. Immunocytochemical analysis of hRGCs, differentiated from hESC-derived fresh **(J)** and banked **(K)** RPCs, at the 16th day of RGC co-expressed tdT fluorescence and immunoreactivities corresponding to PAX6. The proportion of tdT^+^ and PAX6^+^ cells in the culture is given in the graph **(L)**. Immunocytochemical analysis of neurites of RGCs, differentiated from hESC-derived fresh **(M)** and banked **(N)** RPCs, reveals the relative length **(O)** and their complexity **(P)** by the Sholl analysis. qPCR analysis of RGCs, derived from fresh and banked hRPCs, reveals the relative expression of transcripts corresponding to RGC regulators and marker genes **(Q)**. Experiments were carried out in triplicates per group. Scale bars: 20 μm **(B, C, E, F)** and 50 μm **(J, K, M,** **and** **N)**.

**Directed differentiation of banked hRPCs into hRGCs:** Next, we carried out a side-by-side comparison of the efficiency and fidelity of hRGC generation from the fresh and banked hRPCs. The viability of banked RPCs post-thaw was 86.5% ± 3.5% (hiPSC-derived RPCs) and 90.5% ± 2.5% (hESC-derived RPCs). hRPCs were subjected to a stage-specific, chemically defined, and directed differentiation protocol (Teotia et al., 2017a) (Figure 2A). We had demonstrated that the step-wise temporal recruitment of sonic hedgehog (Shh), Notch, fibroblast growth factor (FGF), and transforming growth factor beta (TGFβ) signaling allows native or pluripotent stem cell-derived RPCs to recapitulate the hierarchical regulatory gene expression underlying the initiation, differentiation, and maturation of RGCs, enabling directed acquisition of RGC phenotypes (Parameswaran et al., 2015; Teotia et al., 2017a). The resulting hRGCs were characterized by small soma with extensive processes and defined as RGCs by co-expressing POU4F2 and PAX6 immunofluorescence (hiPSC-derived RGCs) and POU4F2-tdT fluorescence and PAX6 immunofluorescence (hESC-derived RGCs). POU4F2 is also expressed in sensory neurons. However, its expression with PAX6, which is expressed in the retina but not in sensory neurons, defines bonafide RGCs (Xiao et al., 2020). No significant difference was observed in the proportion of POU4F2^+^PAX6^+^ hRGCs (Figures 2B–D) and in the length and complexity (Figures 2E–H) of their neurites between hRGCs derived from fresh and banked hiPSC-RPCs. hRGCs from either source expressed RGC regulatory (*POU4F2 and ISL1*), axon growth-promoting (*SOX11*), and phenotype marker (*SCNG and THY1*) genes with levels of *THY1* significantly higher in hRGCs derived from banked RPCs than those from the fresh ones (Figure 2I). Similarly, no significant difference was observed in the proportion of POU4F2-tDT^+^PAX6^+^ hRGCs generated from fresh and banked hESC-RPCs (Figures 2J–L). The length and complexity of their neurites (except at the distal end of the neurites) did not display a significant difference either (Figures 2M–P). However, when comparing hRGCs derived from hiPSCs and hESC (regardless of the fresh or banked RPCs), the former generated more hRGCs than the latter and the length of their neurites were longer. The latter (Figure 2Q) had more variation in the expression of RGC-specific genes compared to the former. These differences and variations in the gene expression may be attributed to inherent differences between hiPSCs and hESCs and/or the replacement of one of the *POU4F2* alleles by *tdT-THY1.2* in the latter (Sluch et al., 2017). Taken together, the results demonstrated that the fresh and banked RPCs are equivalent in generating hRGCs with similar phenotypes.

**FIGURE 3:**
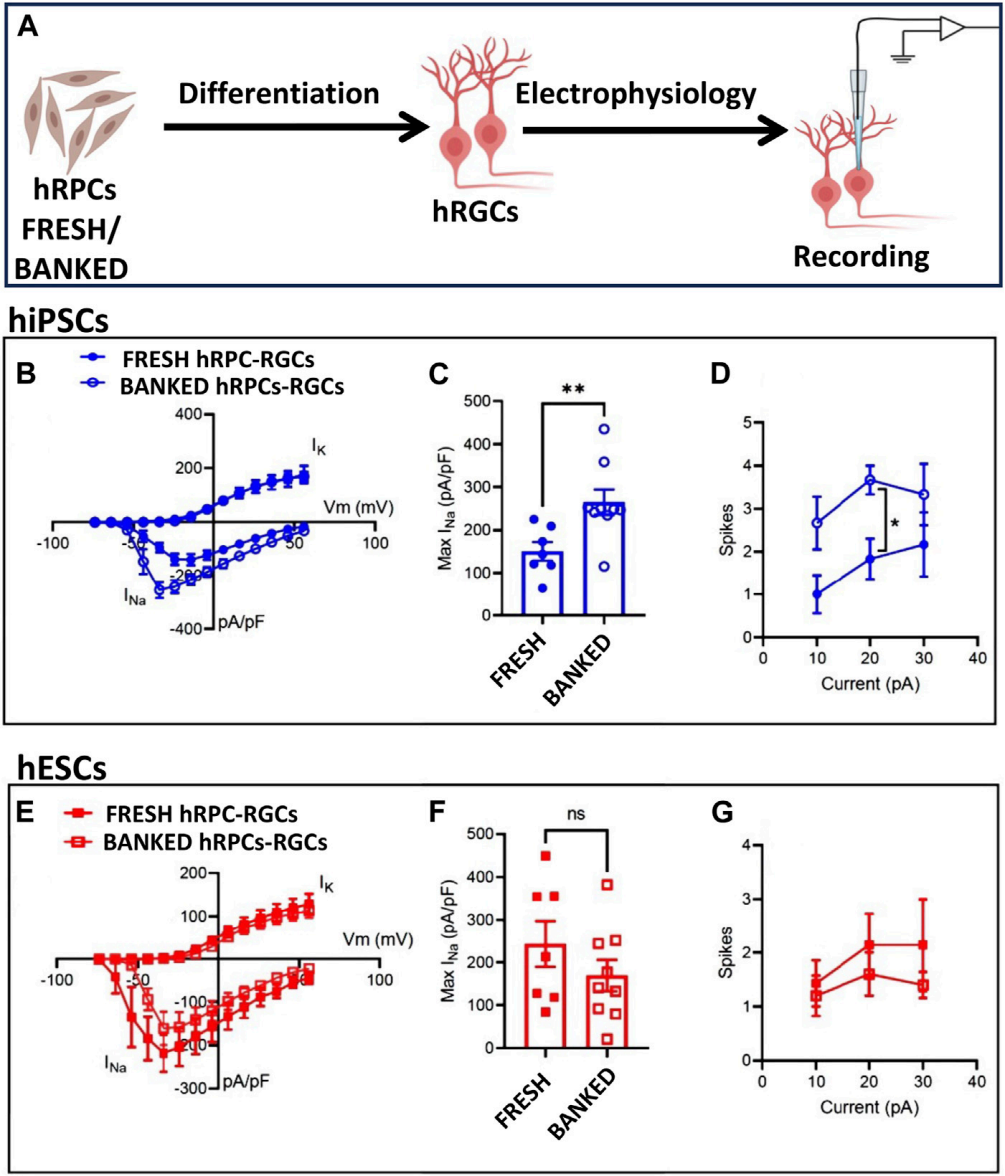
Functional properties of fresh/banked hRPC-derived RGCs. Schematic representation of electrophysiology **(A)**. Measurement of voltage-gated Na^+^ and K^+^ currents in response to depolarizing voltage steps (−74 to +56 mV, 10 mV increments from a holding potential of −84 mV) in hRGCs derived from fresh and banked hiPSC-derived RPCs showing a current–voltage plot of Na^+^ and K^+^ current density **(B)** and peak Na^+^ current density **(C)** (**p < 0.01, unpaired t-test; banked n = 9 cells; and fresh n = 7 cells). Examination of action potential firing in response to depolarizing current injections (500 ms) of hRGCs derived from fresh and banked hiPSC-derived RPCs **(D)**, the former firing more action potentials in response to depolarizing current injections than the latter. Measurement of voltage-gated Na^+^ and K^+^ currents in hRGCs derived from fresh and banked hESC^POU4F2−tdT^-derived RPCs under identical depolarizing voltage steps reveals no significant difference in Na^+^ current density **(E, F)** (banked n = 9 cells and fresh n = 8 cells). No significant difference was observed in action potential firing in response to depolarizing current injections (500 ms) between hRGCs derived from fresh and banked hESC^POU4F2−tdT^-derived RPCs (banked n = 5 cells and fresh n = 7 cells) **(G)**.

**Functional properties of banked hRPC-derived RGCs**: Next, we wanted to know if banked hRPC-derived RGCs have an electrophysiological signature similar to that of RGCs derived from fresh hRPCs, using whole-cell patch-clamp recording (Figure 3A). We first examined RGCs derived from fresh and banked hiPSC-RPCs. Measurement of voltage-gated Na^+^ and K^+^ currents in response to depolarizing voltage steps (−74 to + 56 mV, 10 mV increments from a holding potential of −84 mV; Figure 3B) revealed a small but significant increase in the peak Na^+^ current density (Figure 3C) (Na^+^ current normalized to the whole-cell membrane capacitance, indicating density of Na^+^ channels in the cell membrane) in RGCs derived from banked hiPSC-RPCs than those from fresh hiPSC-RPCs (**p < 0.01, unpaired t-test; banked n = 9 cells; fresh n = 7 cells). Examination of action potential firing in response to depolarizing current injections (500 ms) revealed that RGCs derived from banked hiPSC-RPCs were slightly more excitable than those derived from fresh hiPSC-RPCs, firing more action potentials in response to depolarizing current injections (Figure 3D). There was a statistically significant difference at the 20 pA current injection (*p < 0.05, unpaired t-test; banked n = 6 cells; direct N = 6 cells). Measurement of voltage-gated Na^+^ and K^+^ currents in RGCs under identical depolarizing voltage steps revealed no significant difference in the Na^+^ current density (Figures 3E, F) between RGCs derived fresh and banked hESC-RPCs (banked n = 9 cells: fresh n = 8 cells). Similarly, no significant difference was observed in action potential firing in response to depolarizing current injections (500 ms) between RGCs derived fresh and banked hESC-RPCs (banked n = 5 cells: fresh n = 7 cells) (Figure 3G). A direct comparison of action potential generation revealed that RGCs derived from hiPSC-RPCs were more excitable than hESC-RPC-derived RGCs (data not shown) implicating inherent differences between the pluripotent cells for the observed variations in the functional efficiency of hRGCs. Next, we determined the transplantation, target recognition, and regeneration potential of hRGCs derived from banked hESC-RPCs (henceforth, banked hRPC-derived RGCs), taking advantage of the real-time identification of the cells and their processes using tdT fluorescence. These tests were preceded by determining the transcriptional signature of RGCs derived from banked hRPCs.

**FIGURE 4:**
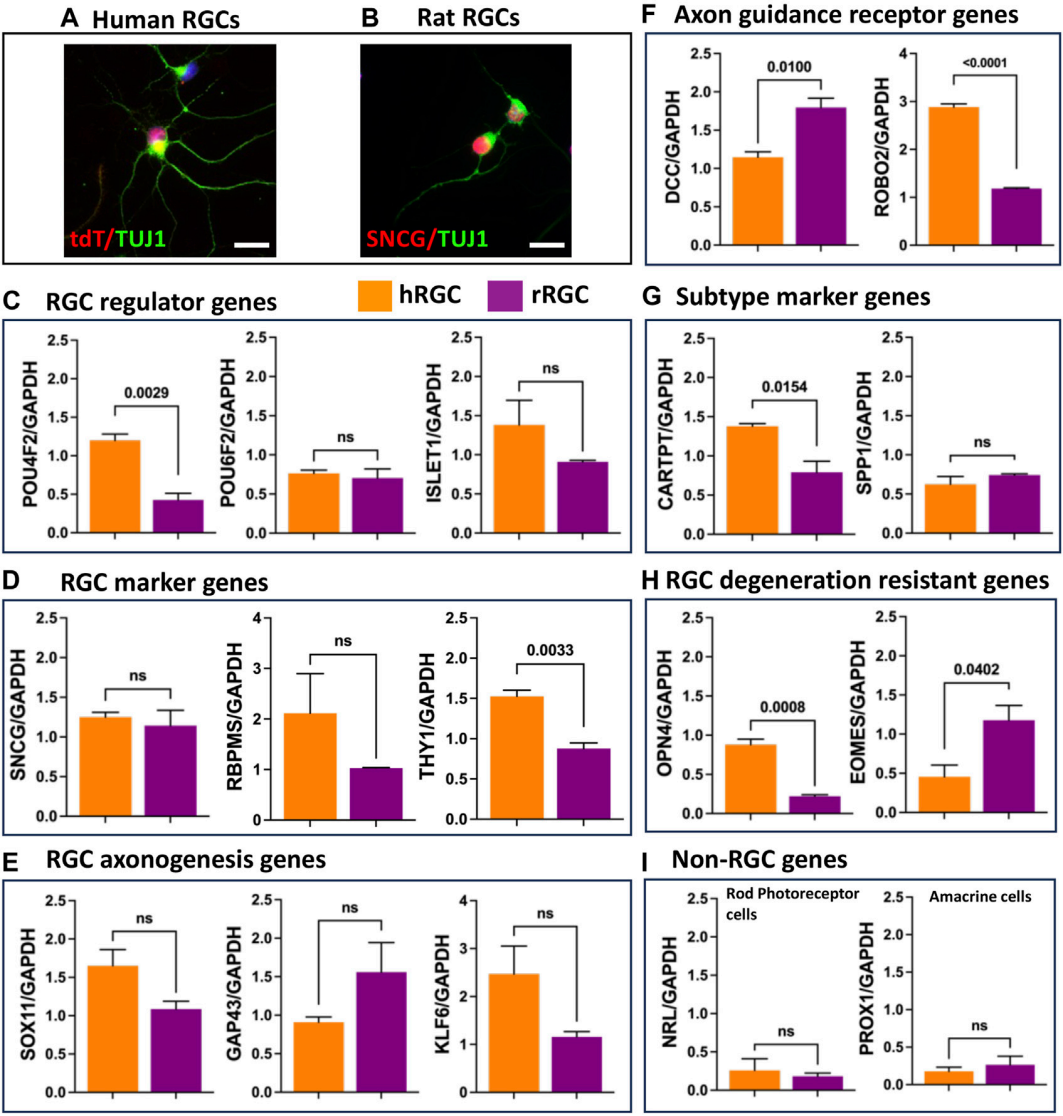
Transcriptional signature of enriched banked hRPC-derived RGCs and native rat RGCs. Immunofluorescence analysis of enriched hRGCs **(A)** and rat RGCs **(B)**. Relative expression levels of the RGC regulator gene **(C)**, RGC marker genes **(D)**, RGC axonogenesis genes **(E)**, RGC axon guidance receptor genes **(F)**, RGC subtype marker genes **(G)**, RGC degeneration resistant genes **(H)**, and non-RGC genes **(I)**. Scale bar: 20 μm.

**Transcriptional signature of banked hRPC-derived RGCs**: To determine the fidelity of RGCs generated from the banked hRPCs, we compared their RGC-specific transcriptomic response to that of native rat retinal RGCs. hRGCs (Figure 4A) and rat RGCs (Figure 4B) were enriched by the immune-panning day 16 RGC culture and PN4 retinal cell dissociates, respectively. We examined the relative expression of transcripts corresponding to RGC regulator genes (*POU4F2*, *POU6F2*, and *ISLET1*) (Figure 4C), RGC marker genes (*SNCG, RBPMS,* and *THY1*) (Figure 4D), RGC axonogenesis genes (*SOX11*, *GAP43*, and *KLF6*) (Figure 4E), RGC axon-guidance receptor genes (*DCC* and *ROBO2*) (Figure 4F), RGC subtype-specific marker genes [*CARTPT* (ON-OFF DSRGCs) and *SPP1* (OFF RGCs)] (Figure 4G), and genes characterizing RGC degeneration-resistant subtype IP-RGCs (OPN4 and EOMES) (Figure 4H), normalized to the expression of housekeeping gene GAPDH in human and rat RGCs (Duan et al., 2015; Teotia et al., 2019; Tran et al., 2019; Ahmad et al., 2020). The qPCR analysis of normalized gene expression revealed that hRGCs shared expression of each of the 15 genes examined with native rat RGCs. There were significant differences in levels of expression of some genes (e.g., *DCC, ROBO2, OPN4, EOMES, CART, and THY1*), which might be species-specific and/or reflecting the relative immaturity of hRGCs (day 16 of the RGC culture) and rat RGCs, derived from PN4 retina. Transcripts corresponding to photoreceptor-specific (*NRL*) and amacrine cell-specific (*PROX1*) genes were detected at very low levels compared to all RGC-related genes (Figure 4I). Together, these observations suggested that RGCs generated from banked hRPCs have acquired the evolutionarily conserved transcriptional code of RGCs.

**FIGURE 5:**
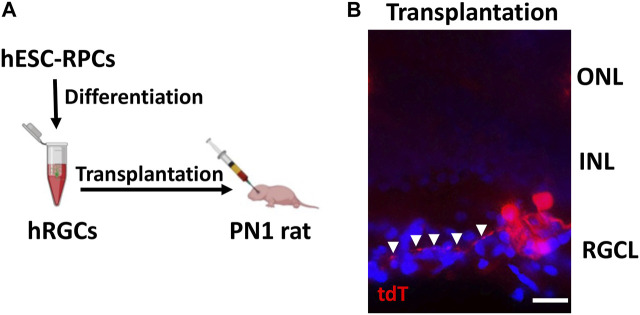
Incorporation of banked hRPC-derived RGCs in the host retina. Schematic of the intravitreal transplantation of banked hRPC-derived RGCs in immunosuppressed PN1 rat pups **(A)**. Transplants were analyzed 14 days post-injection. The transplanted hRGCs, tracked by tdT fluorescence, were observed integrated within the host’s RGC layer and elaborated neurites (arrowheads) **(B)**. Arrowheads show the centripetal trajectory of transplanted cell neurites. Scale bars: 20 μm.

**Transplantation of banked hRPC-derived RGCs**: The *ex vivo* stem cell therapy involves the transplantation of RGCs generated from human pluripotent stem cells, where one of the most important criteria for its success is the survival and lamina-specific incorporation of the transplanted cells followed by their ability to elaborate synapse-forming dendrites and axons that can be guided to the central targets. We had previously tested this approach by transplanting RGCs derived from rodent pluripotent cells in the rat ocular hypertension model of glaucomatous degeneration (Morrison et al., 1997) and observed that the transplanted cells survived and incorporated in the RGC layer of the host retina (Parameswaran et al., 2015). However, the elaboration of neurites by the transplanted RGCs was rudimentary suggesting either deficient neuritogenesis in nascent RGCs or a non-conducive host’s environment or both. To determine whether or not RGCs derived from banked hRPCs possess the fundamental attributes for successful transplantation, i.e., survival, incorporation, and neuritogenesis, we carried out intravitreal transplantation of banked hRPC-derived RGCs in immunosuppressed PN1 rat pups (Figure 5A). Transplanted eyes were examined two weeks later. Examination of the rat retina revealed the majority of transplanted hRGCs, as revealed by tdT fluorescence, at the interface of the retina and lens. However, a minor proportion had integrated within the RGC layer of the host’s retina and survived. More significantly, tdT fluorescence revealed elaboration of neurites by transplanted hRGCs within the host retina, which like that of developing rat RGCs (Brittis and Silver, 1995; Harada et al., 2007) was centripetally oriented away from the peripheral retina (Figure 5B). To determine the possibility of cytoplasmic exchange of tDT between cells (Santos-Ferreira et al., 2016), we carried out immunohistochemical analysis using the STEM121 antibody to detect human-specific cytoplasmic protein from the CNS (Imai et al., 2023). The STEM121 immunoreactivities were exclusively localized with tdT^+^ transplanted cells and not with the tdT^−^ host cells in the RGC layer ruling out the donor–host cytoplasmic exchange-based false positive results (Supplementary Figure S1). These observations suggested that hRGCs derived from banked RPCs display the potential to incorporate within the host’s RGC layers, albeit at low efficiency likely due to the inner limiting membrane (ILM) barrier (Zhang et al., 2021), and elaborate directional neurites.

**FIGURE 6:**
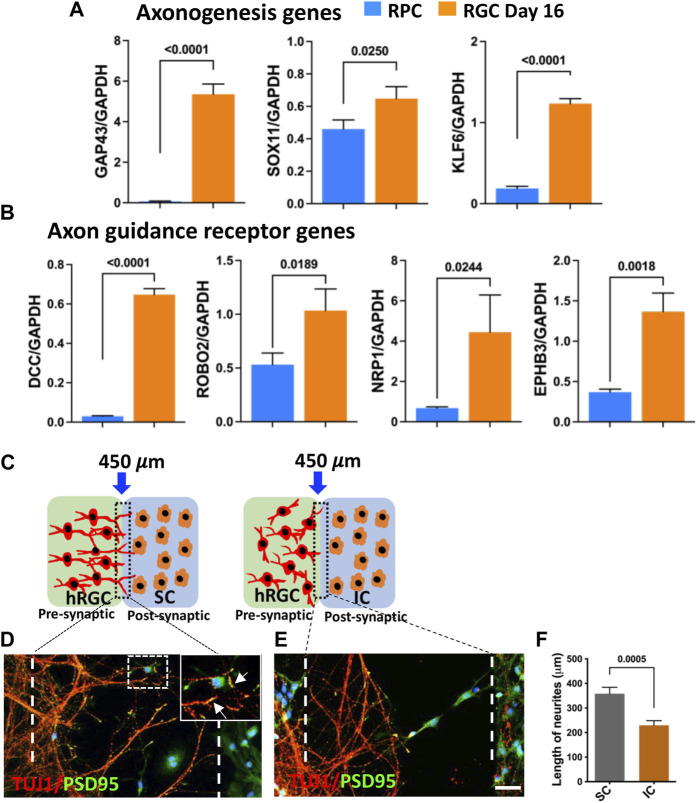
Target recognition by banked hRPC-derived RGCs. Temporal qPCR analysis of gene expression during RGC generation reveals that the differentiation of banked hRPCs into RGCs increases the levels of transcripts corresponding to axon growth-promoting (*GAP43, SOX11, KLF6*) **(A)** and axon guidance receptor (*DCC, ROBO2, NRP1, EPHB3*) genes **(B)**, suggesting the acquisition of the ability of *ex vivo*-generated hRGCs to respond to spatially distributed guidance cues for navigation and target recognition. Schematic representation of hRGCs and the rat colliculus cell co-culture paradigm across a removable silicon barrier (dotted lines) **(C)**. Banked hRPC-derived RGCs and rat SC/IC are cultured in the presynaptic and post-synaptic chamber, respectively. When the barrier is removed, hRGCs neurites, identified by TUJ1 immunoreactivities, orient and extend toward rat SC cells and form synapses, identified by PSD95^+^puncta **(D, F)**. In contrast, neurites elaborated by hRGCs retract in the presence of rat IC **(E, F)**. Experiments were carried out in triplicates per group. Scale bars: 50 μm.

**Target recognition by banked hRPC-derived RGCs**: The centripetal orientation of transplanted cell neurites suggested that they may be able to read the guidance cues for navigation within the retina and recognize central targets (Erskine and Herrera, 2007; Harada et al., 2007). Therefore, we first examined whether or not the banked hRPC-derived RGCs express axon growth-promoting and guidance receptor genes (Parameswaran et al., 2015; Teotia et al., 2019; Ahmad et al., 2020). Temporal analysis of gene expression during the differentiation of RPCs into RGCs revealed the activation of transcripts corresponding to *GAP43*, a cytoplasmic protein essential for guiding axons from the optic chiasm into the optic tracts (Erskine and Herrera, 2007), and those encoding axon growth promoting transcription factors *SOX11* (Norsworthy et al., 2017) and *KLF6* (Moore et al., 2009) (Figure 6A). We also observed temporal activation of transcripts corresponding to *ROBO2*, which facilitates guidance within the retina and at the optic chiasm, *DCC*, which is required for the exit of the axons at the optic disc, neuropilin-1 (*NRP1*), for keeping the axons coalesced, and *EPHs* for establishing the spatial gradient of connections in the superior colliculus (SC) (Erskine and Herrera, 2007; Harada et al., 2007) (Figure 6B). Together, temporal activation of genes for axon growth and guidance suggested that banked hRPC-derived RGCs have acquired molecular properties necessary for axon growth and guidance to reach and discriminate between specific and non-specific targets. We tested this premise in an experimental paradigm where banked hRPC-derived RGCs were co-cultured with cells from the SC across a silicon barrier (Figure 6C). Given the fact that retino-colliculi connections are phylogenetically old (Masterton, 2013), molecules mediating these connections were expected to be evolutionarily conserved and, thus, functional across the species. In a separate set of experiments, banked hRPC-derived RGCs were similarly cultured with inferior colliculus (IC) cells that receive an auditory input (Figure 6C). The removal of the silicon partition led to rich elaboration of hRGC processes toward the SC cells forming synaptic connections (Figure 6D). In contrast, hRGCs elaborated shorter processes that collapsed midway and did not reach IC cells (Figure 6E). Measurement of the length of the processes elaborated by hRGCs revealed a significant increase in their length when cultured in the proximity of SC cells compared to those close to IC cells (Figure 6F). Together, these observations suggested that banked RPC^POU4F2−tdT^-derived hRGCs were molecularly adept for elaborating guidable axons that can discriminate between SC and IC cells.

**FIGURE 7:**
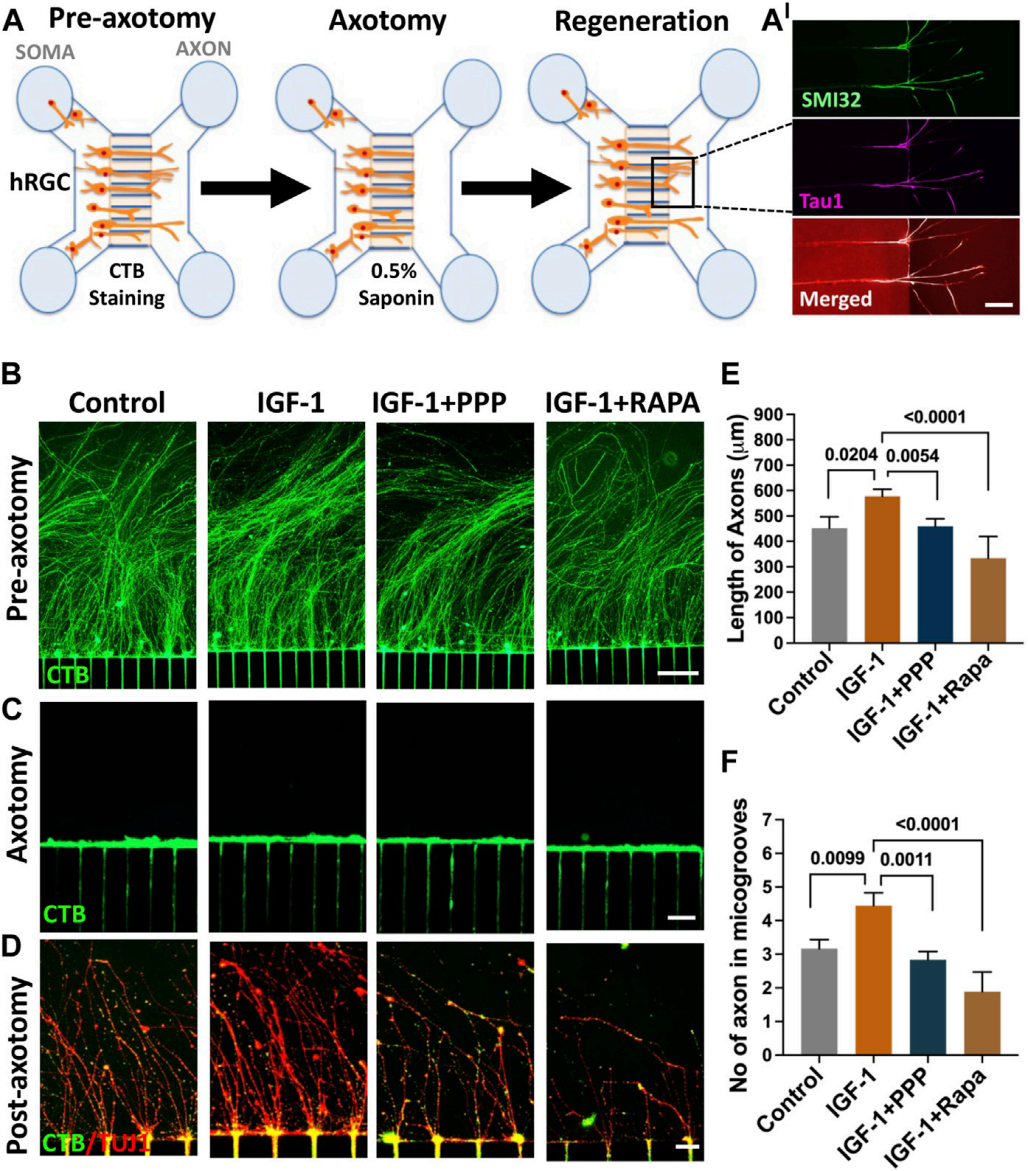
Regeneration by banked hRPC-derived RGCs. The microfluidic model of hRGC axon regeneration following chemical axotomy is depicted schematically **(A)** in the schematic, we show SMI32- and Tau1-positive neurites in the groove, demonstrating that they are axons. Banked hRPC-derived RGCs seeded in the Matrigel-coated soma chamber elaborate axons through the microgrooves into the axon chamber (Teotia et al., 2019). Axons are labeled retrogradely with fluorescent CTB followed by detergent-based chemical axotomy. Regeneration of CTB/TUJ1-positive axons in axon chamber is quantified for their number and length under different culture conditions. The upper panel shows CTB-stained hRGCs axons in the axon chamber in different groups’ pre-axotomy **(B)**. The middle panel shows saponin-mediated axotomy in the axon chamber **(C)**. The lower panels show axon regeneration when cells in the soma chamber are exposed to IGF-1, IGF-1+PPP, and IGF-1+rapamycin post-axotomy *versus* controls **(D)**. Both the length and number of axons increase significantly in the presence of IGF-1 *versus* controls, the effects of IGF-1 abrogated in the presence of PPP, suggesting they are due to the recruitment of the IGF-1R tyrosine kinase pathway **(E, F)**. The facilitatory effects of IGF-1 on the length and number of axons are compromised significantly in the presence of rapamycin suggesting the recruitment of the mTOR axis by IGF-1/IGF-1R signaling for axon regeneration **(E** **and** **F)**. Experiments were carried out in triplicates per group. Scale bars: 100 μm **(B)** 50 μm **(C)** and 20 μm **(D)**.

**IGF-1 mediated axon regeneration in banked hRPC-derived RGCs**: We have previously demonstrated that hiPSC-derived RGCs can regenerate their axons following axotomy and that the underlying mechanism includes mTOR signaling (Teotia et al., 2019), as observed in the optic nerve crush (ONC) model (Park et al., 2008). We examined whether or not RGCs derived from banked hRPCs possess the regenerative potential displayed by those derived from fresh hRPCs (Teotia et al., 2019) and test the premise that the IGF-1/IGF-1R pathway is evolutionarily conserved in hRGCs for facilitating axon regeneration. The permissive role of the IGF-1/IGF-1R pathway in optic nerve regeneration has been demonstrated in fish (Koriyama et al., 2007) and mice (Duan et al., 2015; Liu et al., 2017; Zhang et al., 2019). The premise was tested in a microfluidic model of axon regeneration established in our lab (Teotia et al., 2019), in which banked hRPC-derived RGCs, seeded in the soma chamber of a microfluidic device, elaborated axons (SMI32^+^Tau1^+^ neurites) through the microgrooves to enter the axon chamber (Figures 7A, A’) Axons in the axon chamber were retrogradely labeled with CTB-488 (Figure 7B), before being subjected to saponin-based chemical axotomy (Figure 7C), to identify regenerating axons by co-labeling with CTB-488 staining and TUJ1 immunofluorescence (Figure 7D). Axon regeneration was examined post-axotomy following separate groups: untreated control, IGF-1 treatment, IGF-1+ picropodophyllin (PPP) treatment, and IGF-1+rapamycin treatment groups. Axon regeneration was observed within 48 h in all groups including controls as previously observed in hiPSC-derived RGCs (Teotia et al., 2019). Quantification of regeneration of CTB-488^+^ TUJ1^+^ axons was carried out on the 5th day post-axotomy, which revealed that their length (Figures 7D, E) and number (Figures 7D–F) in the axon chambers were significantly higher in banked hRPC-derived RGCs exposed to IGF-1 *versus* controls (axon number/microgroove: 3.167 ± 0.428 *versus* 4.44 ± 0.467, P=<0.009; axon length: 452.2 ± 52.16 μM *versus* 577.7 ± 52.16 μM, P=<0.02). Co-exposure of cells to IGF-1 and PPP, an inhibitor of IGF-1R tyrosine kinase (Menu et al., 2006; Xia et al., 2018), significantly decreased the indices of axon regeneration when compared to regeneration observed in the presence of IGF-1 alone (axon number/microgroove: 2.83 ± 0.845 *versus* 4.44 ± 0.467, P=<0.001; axon length: 459.4 ± 63.88 μM *versus* 577.7 ± 52.16 μM, P=<0.005), demonstrating the specificity of the recruitment of the IGF-1/IGF-1R pathway by the axotomized cells during axon regeneration (Figures 7D–F). To know if the IGF-1-mediated axon regeneration involved the mTOR axis (Duan et al., 2015), we carried out IGF-1-mediated regeneration in the presence of rapamycin, a specific inhibitor of the mTORC1 complex (Lamming, 2016). Rapamycin blocked the IGF-1 mediated regeneration (axon number/microgroove: 1.88 ± 0.405 *versus* 4.44 ± 0.467, P = <0.0001; axon length: 333.8 ± 33.08 μM *versus* 577.7 ± 52.16 μM, P = <0.0001) (Figures 7D–F), suggesting that IGF-1/IGF-1R recruits the mTOR axis to promote axon regeneration in axotomized cells. Together, these results suggested that the banked hRPC-derived RGCs possess regeneration potential and represent a facile and reliable source for examining the evolutionarily conserved mechanism underlying axon regeneration, which can be targeted for therapeutics.

## Discussion

The mechanism underlying selective and universal degeneration of RGCs in glaucoma remains poorly understood, and strategies to rescue glaucomatous degeneration remain elusive (Teotia et al., 2017c). Recent advancement in cell reprogramming and gene editing technologies has posited human pluripotent cells, hiPSCs and hESCs, as a practical approach for modeling glaucoma to understand the underlying mechanism of degeneration and generating hRGCs for *ex vivo* stem cell therapy to replace degenerating RGCs (Ahmad et al., 2020). Both disease modeling and *ex vivo* stem cell approaches require the reproducible induction of pluripotent cells along the retinal lineage and directed and efficient differentiation of the resulting RPCs into RGCs with phenotype, function, and capacity for guided interactions with their targets like their native counterparts. In addition, development of a method that is rapid while preserving the efficiency and fidelity of hRGC generation is desirable from the perspective of supporting a rapid turnaround of disease modeling experiments and availability of hRGCs for practical clinical utilization.

The efficiency of hRGC generation directly depends on the efficiency of the retinal induction of the human pluripotent stem cells (Ahmad et al., 2020). There are two approaches for the retinal induction of pluripotent stem cells based on the developmental principle, one that taps into the passive manifestation of the default neural potential of the embryonic ectoderm (Munoz-Sanjuan and Brivanlou, 2002; Pankratz et al., 2007) for generating RPCs (Zhao et al., 2002; Meyer et al., 2009) and the other that actively recruits the neural potential by influencing the underlying pathways through small molecules and growth factors (Tropepe et al., 2001; Aubert et al., 2002; Ying et al., 2003; Pera et al., 2004; Lindsley et al., 2006) to generate RPCs (Lamba et al., 2006; Osakada et al., 2009; Lamba et al., 2010; Parameswaran et al., 2010; Parameswaran et al., 2015). We had previously adapted the latter approach for the generation of hRPCs, which had two significant drawbacks. First, the induction process was long, and second and more importantly, the reproducibility of RPC generation varied from person-to-person skills in selecting EBs for NR formation. The adaptation of the dual SMAD inhibitor monolayer culture neural induction protocol (Chambers et al., 2009) with retinal differentiation through the inhibition and activation of Wnt and IGF-1 signaling, respectively, reduced the duration of the protocol from 28 days to 9 days and made it reproducible and bankable. The efficiency of the generation of RPCs from either hiPSCs or hESCs is in excess of ∼80%, as determined by proliferating cells co-expressing RX and Pax6 immunoreactivities. The retinal induction is a regulated process demonstrated by temporal silencing of pluripotent and germ-layer-specific genes and activation of EFTF genes. The regulated activation of the RGC phenotype ensured phenotype stability.

The banked hRPCs can be thawed to a high survival rate in the presence of ROCK inhibitor and directly differentiated into hRGCs using the method that recapitulates the developmental mechanism, divided into three phases (Teotia et al., 2017a; Ahmad et al., 2020): initiation, differentiation, and maturation. Signaling pathways underlying each of these stages were recruited using small molecules and/or recombinant ligands. For example, it has been observed that transient FGF signaling by FGF8 and FGF3 facilitates initial RGC differentiation in the central retina (McCabe et al., 1999; Martinez-Morales et al., 2005). Shh is thought to be similarly involved. However, beyond the initiation of RGC differentiation, Shh may promote proliferation and, thus, maintain RPCs for subsequent differentiation (Zhang and Yang, 2001; Martinez-Morales et al., 2005). A coordinated decrease in Notch signaling is essential for RPCs to commit along the RGC lineage (James et al., 2004; Nelson et al., 2006). Based on these observations, transient exposure of hRPCs to FGF8 and Shh signaling and simultaneously inhibiting Notch activities initiate RGC specification. Differentiation is promoted by keeping Notch activities and inhibiting TGFβ signaling. The latter is important given the observation that the activation of the TGFβ pathway by GDF11, secreted by differentiating RGCs, inhibits RGC differentiation (Kim et al., 2005). The survival of nascent RGCs depends on neurotrophins to prevent the activation of programmed cell death (PCD) (Guerin et al., 2006). Thus, to facilitate RGC maturation without excessive cell death, BDNF, NT4, and CNTF, all known to prevent PCD in RGCs (Cohen-Cory and Fraser, 1995; Meyer-Franke et al., 1995; Ji et al., 2004; Spalding et al., 2004), are included with general promoters of cell survival, forskolin, and ROCK inhibitor (Meyer-Franke et al., 1995; Lingor et al., 2008; Parameswaran et al., 2015; Teotia et al., 2017a). This led to recapitulation of the hierarchical expression of RGC regulators, generating RGCs with a similar efficiency between fresh or banked RPCs, derived from either hiPSCs or hESCs. More significantly, RGCs derived from banked hRPCs demonstrated a similar phenotype, neurite length, and complexity, as those differentiated from freshly generated RPCs, regardless of their hiPSC or hESC origin. The physiological response between RGCs derived from fresh and banked hESC-derived RPCs were similar as opposed to those generated from iPSC-derived RPCs, which for the latter may be due to a specific iPSC line. The variations in physiological response between hESCs and hiPSC-derived RGCs may be due to the difference in the lineage of these cells, the latter retaining some of the epigenetic signature of the parental somatic cells. Their reproducibility of generating the RGC phenotype and function across different human pluripotent stem cells with a transcriptional signature similar to that of native RGCs posit the banked RPCs suitable for disease modeling, which involves different patient-specific iPSCs with different mutations, polymorphisms, and controls ranging from healthy donor to gene-edited isogenic controls. Their utility in supporting *ex vivo* stem cell approaches to glaucomatous degeneration is demonstrated by their molecular make up for axon growth and guidance and their ability to discriminate between specific (SC) and non-specific (IC) central targets, the essential features required of the *ex vivo* generated hRGCs to functionally replace degenerated RGCs. The elaboration of directional neurites by the transplanted hRGCs represents preliminary evidence of their ability to read the evolutionarily conserved guidance cues within the host retina for exit at the optic nerve head (Brittis and Silver, 1995; Ahmad et al., 2023). The limitation appears to be the efficient incorporation of the transplanted hRGCs in the host retina, which might be due to the physical and chemical barrier offered by the ILM, which may be addressed through enzymatic treatment (Zhang et al., 2021) or neutralization of chemo-repulsive molecules (Ahmad et al., 2023).

We had previously demonstrated that mTOR signaling regulates the differentiation of hRGCs from hiPSCs and that recapitulation of this pathway supported the regeneration of hRGC axons following axotomy in a microfluidic model of axon regeneration (Teotia et al., 2019). We tested the regenerative potential of hRGCs derived from the banked hRPCs in the context of the IGF-1/IGF-1R signaling pathway, which has been demonstrated to play an important role in axonal specification and growth in hippocampal pyramidal neurons (Sosa et al., 2006), corticospinal motor neurons (Özdinler and Macklis, 2006), and RGCs (Dupraz et al., 2013). The observation that the IGF-1/IGF-1R signaling pathway facilitates optic nerve regeneration in goldfish (Koriyama et al., 2007) suggested that the recapitulation of this pathway may support optic nerve regeneration in higher vertebrates. Accordingly, the role of the IGF-1/IGF-1R signaling pathway in optic nerve regeneration was demonstrated in the ONC model in mice (Duan et al., 2015; Liu et al., 2017; Zhang et al., 2019). Our results demonstrate that IGF-1/IGF-1-R-mediated optic nerve regeneration is evolutionarily conserved in hRGCs, which can be reproducibly examined using banked hRPCs. However, unlike the observation in the ONC model (Duan et al., 2015), IGF-1 alone was able to promote axon regeneration in hRGCs through the recruitment of the mTOR pathway. Our observations support the notion that the mTOR pathway represents one of the major axes through which the intra-cellular response to factors for promoting axon regeneration is coordinated. In summary, we have demonstrated a rapid method of directed generation RGCs, derived from banked human pluripotent stem cell-derived RPCs by recapitulation of developmental mechanism(s), which may be suitable for versatile disease modeling and identification of pathways for therapeutic regeneration, and practically support clinical *ex vivo* stem cell approach to glaucomatous degeneration.

## Materials and methods

**Experimental animals:** Experimental protocols and the use of animals were approved by the Institutional Animal Care and Use Committee, at the University of Nebraska Medical Center (UNMC), and conducted in accordance with the Association for Research in Vision and Ophthalmology (ARVO) Statement for the use of animals in ophthalmic and vision research. Timed-pregnant Sprague Dawley rats (Charles River Laboratories) were used as SC/IC cell donors from PN1/PN3 pups.

**Neural/retinal induction:** For neural/retinal induction, pluripotent stem cells were dissociated with accutase (Gibco) 1 ml/well for 10 min at 37°C and suspended in the freshly prepared neural induction media [(NIM); 1% B27 supplement (Gibco), 1% N2 supplement (Gibco), 10 µM SB431542 (Stemgent), 100 nM LDN193189 (Stemgent), and 10 µM ROCK inhibitor (Milteny Biotech) in 12 media (Gibco)] and plated at a density of 2 × 10^6^ cells/10 cm^2^ on Matrigel (Corning)-coated culture plates. Culture was continued for 3 days with daily media change without ROCK inhibitor (Milteny Biotech) after the 1st day. At the 3rd day of the culture, the NIM was changed to retinal induction media [(RIM); 1% B27 supplement (Gibco), 1% N2 supplement (Gibco), 10 ng/ml hDKK1 (R&D system), 10 ng/ml IGF-1 (R&D system), and 100 nM LDN193189 (Stemgent)] in DMEM:F12 media (Gibco), and culture was continued for another 6 days with daily media change. Cells were collected at different time points for temporal immunocytochemical and qPCR analyses.

**Banking of hRPCs:** Freshly generated RPCs at the 9^th^ day of culture were dissociated into single cells in accutase (Gibco) 1 ml/well for 15 min at 37°C, followed by mild trituration. Cells were counted by hemocytometer and 10^7^ cells centrifuged at 3000 rpm for 3 min at RT. RPC pellets were suspended in 2 ml of neural progenitor freezing medium (STEMCELL Technologies) at a cell density of 5 × 10^6^ cells/ml. Suspended cells were subjected to slow freezing in 99.9% isopropanol (ACROS organics) at −80°C overnight. The next day cells were transferred to a liquid N_2_ tank for long term banking.

**Differentiation of hRPCs into hRGCs:** Fresh/banked hRPCs were thawed in 37°C water bath for 5 min and transferred to a fresh Falcon tube containing 5 ml of DMEM: F12 and centrifuged at 1000 rpm for 3 min at RT. Cell pellets were suspended in 2 ml of RIM with 10 µM ROCK inhibitor (Milteny Biotech) and 1 ml of cell suspension were plated per well of a Matrigel-coated 6-well plate and incubated for 2 h at 37°C for the cells to adhere to the plate. Each well was supplemented with 1 ml of RIM with 10 µM ROCK inhibitor (Milteny Biotech) and incubation was continued at 37°C overnight. Next day, media was replaced by fresh RIM without ROCK inhibitor before continuing the culture for two more days by which time cells were ready for RGC differentiation. hRPC differentiation into functional hRGCs was performed using a chemically defined medium following our established protocol (Teotia et al., 2017a). Then, RGC differentiation was initiated by treating cells for 3 days with 250 ng/ml Shh (R&D System), 100 ng/ml FGF8 (R&D System), 100 nM LDN (Stemgent), and 3 μM DAPT (Sigma). RGC differentiation was facilitated by treatment with 100 μg/ml follistatin (R&D System), 250 ng/ml Shh (R&D System), 100 nM LDN (Stemgent), and 3 μM DAPT (Sigma) for 3 days. Finally, RGC maturation and survival were promoted by supplementing the medium with 100 ng/ml BDNF (R&D System), 10 μM forskolin (R&D System), 5 ng/ml NT4 (R&D System), 10 ng/ml CNTF (R&D System), 400 μM cAMP (STEMCELL Technologies), 10 μM ROCK inhibitor (Milteny Biotech), and 3 μM DAPT (Sigma) for the next 10 days. Media were changed every day. The factors for hRGC initiation, differentiation, and maturation were dissolved in the basal RGC medium (DMEM:F12/neuro basal medium at 1:1 ratio with N2 supplement (0.05%), B27 supplement (0.5%), L-glutamine (5%), β−mercaptoethanol (1%), BSA (1 μg/ml), insulin (5 μg/ml), sodium selenite (3 nM), and transferrin (50 μg/ml). All reagents were purchased from R&D Systems, Sigma-Millipore, and GIBCO-Thermo Fisher Scientific.

**Immune-panning of human and rat RGCs**: Enrichment of hRGCs/rat RGCs by immune-panning was carried out as previously described (Meyer-Franke et al., 1995; Winzeler and Wang, 2013). In brief, retinal cells from postnatal day 4 (PN4) Sprague Dawley rats and banked hESC/RPC-derived day 16 RGC culture were dissociated using the papain dissociation system (Worthington Biochemical Corporation) as per the manufacturer’s instruction. The dissociated cells were serially immune-panned over negative panning dishes to remove macrophage [goat anti-mouse IgG antibody-coated dish to remove anti-mouse macrophage mAB (Sigma) bound macrophages] and amacrine cells [HNK/N-CAM (Sigma)-coated dish]. The macrophage- and amacrine cell-depleted cell dissociates were transferred to positive panning dishes of anti-mouse CD 90.1 (Thy1.1; Bio-rad) and anti-mouse CD90.2 (Thy1.2; Millipore) to enrich rat and banked hRPC-derived RGCs, respectively. Floating cells were removed by washes, and adherent enriched RGCs were recovered by trypsin digestion. The enrichment of banked hRPC-derived RGCs against the Thy1.2 antigen was possible due to its expression under the POU4F2 promoter, engineered in the parental hES cells (Sluch et al., 2017).

**Microfluidic model of hRGC axon regeneration:** Axon regeneration was examined using the microfluidic device as previously described (Teotia et al., 2019; Ahmad et al., 2020). In brief, polydimethylsiloxane microfluidic devices with 450 μm microgrooves (SND 450, Xona Microfluidics) were assembled and prepared as per the manufacturer’s instruction. Then, cleaned, sterilized, and dry devices were reversibly attached to a poly-D-lysine-coated cover glass (corning, 50 × 24 mm) by applying gentle force to seal them for axon outgrowth. Once assembled, the soma and axon chambers were coated with 1% Matrigel (Corning) in DMEM:F12 medium (Invitrogen) for 1 h at room temperature before plating of 80,000 cells in hRGC growth media in the soma chamber. The axon chamber was filled with similar media to facilitate axonal growth. Volumes in the wells were adjusted to ensure flow of media from the soma (200 μl per well) to the axon (150 μl per well) chamber. Media in the devices were changed every 2 days. Retrograde labeling (between the 5th and 6th day of culture) of axons and soma was performed by adding 1% cholera toxin subunit B (CTB) Alexa Fluor 488 conjugate (Thermo Fisher) dissolved in hRGC media to the axon chamber (100 μl per well) of the microfluidic device. After overnight incubation at 37°C, the axon chamber’s media were removed, the chamber was rinsed, and axotomy was performed adding 50 μl RGC medium with 0.5% saponin (Sigma) for 3 min at 37°C in an incubator. To prevent the flow of the detergent into the soma compartment, a hydrostatic pressure was maintained by volume difference between soma (200 μl/well) and axon (50 μl/well) chambers. At the end of the 3 min period, followed by two PBS washes, the axon chamber was re-coated with 1% Matrigel for 45 min at 37°C in an incubator. After Matrigel coating, media were returned immediately to the axonal chamber for the duration of the culture time. In separate groups, hRGC media post-axotomy was supplemented with 10 ng/ml IGF-1 (R&D system), 10 ng/ml IGF-1 + 500 nM PPP (Sigma), and 10 ng/ml IGF-1 + 100 nM rapamycin (Sigma), to detect the specificity of IGF-1/IGF-1R signaling and IGF-1-mediated recruitment of the mTOR axis, respectively.

**hRGCs: colliculus cell co-culture:** Colliculus cell co-culture was performed by modifying a previously described method (Parameswaran et al., 2015; Teotia et al., 2017a). Superior/inferior colliculi, dissected from the brains of PN1/PN3 Sprague Dawley rats, were dissociated using 0.25% trypsin (Gibco) at 37°C for 30 min, followed by gentle trituration. Cell suspensions were centrifuged at 1000 rpm for 3 min, and 2.5 × 10^4^ cells were plated in one of the wells of the two-well silicon insert (IBIDI) fixed over a PDL-coated glass coverslip and coated with 1% Matrigel. RGCs (2.5 × 10^4^) were plated in the other well across a silicon partition. Both superior/inferior colliculi cells were cultured in the basal retinal culture (RCM) medium (Parameswaran et al., 2015) at 37°C for 24 h. After 24 h when the cells were attached to the coverslip, the dual chamber silicon device was removed, and co-culture was continued for another 3 days to allow hRGC neurites to interact with SC/IC cells. Cells were fixed in 4% paraformaldehyde and subjected to immunofluorescence analysis.

**Transplantation of hRGCs**: Retinal transplantation of hRGCs was carried out as described previously (Parameswaran et al., 2015). In brief, PN1 rat pups were anesthetized on ice, and 10,000 hRGCs/μl in RGC growth medium were injected intravitreally using a glass micropipette. Pups were recovered from anesthesia on a warm plate before returning to the mother. Pups were given a daily intraperitoneal injection of cyclosporin (10 mg/kg body weight) and euthanized 14 days post-transplantation. Eyes were enucleated, fixed in 4% paraformaldehyde, frozen in the OCT embedding medium, and cryo-sectioned for tracking and immunocytochemical analysis of transplanted cells.

**Immunocytochemical analysis:** The immunocytochemical analysis was performed as described previously (Teotia et al., 2017a). In brief, paraformaldehyde-fixed cells, exposed to 5% normal goat/donkey serum in PBS for 30 min at room temperature, were permeabilized with 0.1%/0.2%/0.4% Triton X-100 (depending on the sub-cellular location of the antigens), followed by an overnight incubation with the primary antibody at 4°C. A list of antibodies and working dilutions is provided in Supplementary Table S1. Human-specific antibodies and dilution were similar to that described previously (Teotia et al., 2017a; Teotia et al., 2019). Cells incubated with fluorescence (Cy3/FITC)-tagged secondary antibodies were counterstained with DAPI and then used for microscopic visualization. Fluorescent images were acquired using the Zeiss ApoTome Imager M2 upright microscope (Axiovert 200M), and Axiovision 4.8 software was used for image processing (Carl Zeiss, GmbH, Germany, http://www.zeiss.co.in). The percentage of cells expressing specific markers was determined by counting immune-positive cells per total DAPI-positive cells in five randomly selected visual fields per coverslip. The means and standard deviation were calculated from three different experiments. The Sholl analysis was performed with the software ImageJ using the plugin Sholl Analysis (v1.50) with a 20 μm ring interval from neural rosettes, as described previously (Teotia et al., 2017a).

**Quantitative polymerase chain reaction analysis:** The quantitative polymerase chain reaction (Q-PCR) analysis was carried out as described previously (Teotia et al., 2017a; Teotia et al., 2019). Total RNA from cells was extracted using a Mini-RNeasy kit (Qiagen, Inc., Valencia, CA, https://www.qiagen.com) according to the manufacturer’s instructions. 5 μg of total RNA per sample was used for reverse transcription into cDNA synthesis using the Superscript III RT kit, following the manufacturer’s instruction. Q-PCR was performed using Quantifast SYBR Green Master Mix (Qiagen) on Rotor Gene 6000 (Corbett Robotics, San Francisco, CA http://www.corbettlifesciencecom/). The Primer sequences specific to human/rat genes are listed in Supplementary Tables S2, S3. Primer sequences specific to mouse/rat genes were similar to those described previously (Parameswaran et al., 2015). The reaction was performed in triplicates, and results normalized to internal endogenous GAPDH expression.

**Electrophysiological recordings:** Coverslips were affixed to a recording chamber on the stage of an Olympus BX51-WI microscope using vacuum grease and superfused with Ames’ medium (US Biologicals) bubbled with 95% O_2_/5% CO_2_ at room temperature. Cells were targeted for whole-cell patch-clamp recording with pipettes pulled from thin-walled borosilicate glass capillary tubes (1.2 mm OD, 0.9 mm ID) filled with a solution comprised of (in mM) 120 potassium-gluconate, 8 KCl, 2 EGTA, 10 HEPES, 5 ATP-Mg, 0.5 GTP-Na_2_, 5 phosphocreatine-Na_2_, and 0.1 lucifer yellow-Li. Pipettes had resistances of 5–8 MW. Cells were voltage-clamped at −84 mV (after correction for 14 mV liquid junction potential), and voltage-gated Na^+^ and K^+^ currents were recorded in response to a series of depolarizing voltage steps (150 ms, −74 to +56 mV, 10 mV increments). Series resistance was partially compensated (65%–75%). Spiking activity was recorded in response to a series of depolarizing current injections (+2.5 to +15 pA, 2.5 pA increments, 500 ms). Current- and voltage-clamp stimuli were controlled with a Multiclamp 700B amplifier and digitized with a Digidata 1550B. For analysis, Na^+^ and K^+^ currents were normalized to cell capacitance measured using the amplifier circuitry, and I_Na_ was measured at the peak of the current while I_K_ was measured at the end of the 150 ms stimulus step.

**Statistical analysis:** The data were analyzed and plotted using GraphPad Prism (Graphpad, La Jolla CA (http://www.graphpad.com), and Windows Excel (Microsoft, Redmond, United States). Statistical significance was calculated by either an unpaired Student’s t-test (two-tailed) or by one-way analysis of variance (ANOVA) for multiple groups. Statistical differences of *p*-values less than 0.05 were considered significant. All results were obtained from three replicate samples in two independent experiments.

## Data Availability

The original contributions presented in the study are included in the article/Supplementary Material; further inquiries can be directed to the corresponding author.

## Supplementary material

SUPPLEMENTARY FIGURE S1 Evidence against donor–host cytoplasmic transfer. Banked hRPC-derived RGCs were transplanted intravitreally in PN1 rat pups and analyzed 14 days post-injection. The transplanted hRGCs, tracked by tdT fluorescence, were observed integrated within the host’s RGC layer (arrows) **(A, B)**. The incorporated tdT^+^ hRGCs (arrows) and host cells (arrowheads) expressed immunoreactivities corresponding to the human-specific cytoplasmic antigen, recognized by the STEM121 antibody **(C, D)**. Scale bars: 50 μm.

## References

[B1] Ahmad I. Teotia P. Erickson H. Xia X. (2020). Recapitulating developmental mechanisms for retinal regeneration. Prog. Retin Eye Res. 76, 100824. 10.1016/j.preteyeres.2019.100824 31843569 PMC12866890

[B2] Ahmad I. Van Hook M. Subramani M. A. (2023). Human Retinal ganglion cells respond to evolutionarily conserved chemotrophic cues for intraretinal guidance and regeneration. USA: ARVO New Orlean. 10.1093/stmcls/sxad061 PMC13032126 37591511

[B3] Almasieh M. Wilson A. M. Morquette B. Cueva Vargas J. L. Di Polo A. (2012). The molecular basis of retinal ganglion cell death in glaucoma. Prog. Retin Eye Res. 31, 152–181. 10.1016/j.preteyeres.2011.11.002 22155051

[B4] Aubert J. Dunstan H. Chambers I. Smith A. (2002). Functional gene screening in embryonic stem cells implicates Wnt antagonism in neural differentiation. Nat. Biotechnol. 20, 1240–1245. 10.1038/nbt763 12447396

[B5] Brittis P. A. Silver J. (1995). Multiple factors govern intraretinal axon guidance: A time-lapse study. Mol. Cell Neurosci. 6, 413–432. 10.1006/mcne.1995.1031 8581313

[B6] Chambers S. M. Fasano C. A. Papapetrou E. P. Tomishima M. Sadelain M. Studer L. (2009). Highly efficient neural conversion of human ES and iPS cells by dual inhibition of SMAD signaling. Nat. Biotechnol. 27, 275–280. 10.1038/nbt.1529 19252484 PMC2756723

[B7] Chow R. L. Lang R. A. (2001). Early eye development in vertebrates. Annu. Rev. Cell Dev. Biol. 17, 255–296. 10.1146/annurev.cellbio.17.1.255 11687490

[B8] Cohen-Cory S. Fraser S. E. (1995). Effects of brain-derived neurotrophic factor on optic axon branching and remodelling *in vivo* . Nature 378, 192–196. 10.1038/378192a0 7477323

[B9] Duan X. Qiao M. Bei F. Kim I. J. He Z. Sanes J. R. (2015). Subtype-specific regeneration of retinal ganglion cells following axotomy: Effects of osteopontin and mTOR signaling. Neuron 85, 1244–1256. 10.1016/j.neuron.2015.02.017 25754821 PMC4391013

[B10] Dupraz S. Grassi D. Karnas D. Nieto Guil A. F. Hicks D. Quiroga S. (2013). The insulin-like growth factor 1 receptor is essential for axonal regeneration in adult central nervous system neurons. PLoS One 8, e54462. 10.1371/journal.pone.0054462 23349896 PMC3548777

[B11] Eiraku M. Takata N. Ishibashi H. Kawada M. Sakakura E. Okuda S. (2011). Self-organizing optic-cup morphogenesis in three-dimensional culture. Nature 472, 51–56. 10.1038/nature09941 21475194

[B12] Elkabetz Y. Panagiotakos G. Al Shamy G. Socci N. D. Tabar V. Studer L. (2008). Human ES cell-derived neural rosettes reveal a functionally distinct early neural stem cell stage. Genes Dev. 22, 152–165. 10.1101/gad.1616208 18198334 PMC2192751

[B13] Erskine L. Herrera E. (2007). The retinal ganglion cell axon's journey: Insights into molecular mechanisms of axon guidance. Dev. Biol. 308, 1–14. 10.1016/j.ydbio.2007.05.013 17560562

[B14] Guerin M. B. Mckernan D. P. O'Brien C. J. Cotter T. G. (2006). Retinal ganglion cells: Dying to survive. Int. J. Dev. Biol. 50, 665–674. 10.1387/ijdb.062159mg 17051476

[B15] Harada T. Harada C. Parada L. F. (2007). Molecular regulation of visual system development: More than meets the eye. Genes Dev. 21, 367–378. 10.1101/gad.1504307 17322396

[B16] Imai R. Tamura R. Yo M. Sato M. Fukumura M. Takahara K. (2023). Neuroprotective effects of genome-edited human iPS cell-derived neural stem/progenitor cells on traumatic brain injury. Stem Cells 41, 603–616. 10.1093/stmcls/sxad028 37029780 PMC10267696

[B17] James J. Das A. V. Rahnenführer J. Ahmad I. (2004). Cellular and molecular characterization of early and late retinal stem cells/progenitors: Differential regulation of proliferation and context dependent role of Notch signaling. J. Neurobiol. 61, 359–376. 10.1002/neu.20064 15452852

[B18] Ji J. Z. Elyaman W. Yip H. K. Lee V. W. Yick L. W. Hugon J. (2004). CNTF promotes survival of retinal ganglion cells after induction of ocular hypertension in rats: The possible involvement of STAT3 pathway. Eur. J. Neurosci. 19, 265–272. 10.1111/j.0953-816x.2003.03107.x 14725620

[B19] Kim J. Wu H.-H. Lander A. D. Lyons K. M. Matzuk M. M. Calof A. L. (2005). GDF11 controls the timing of progenitor cell competence in developing retina. Science 308, 1927–1930. 10.1126/science.1110175 15976303

[B20] Koriyama Y. Homma K. Sugitani K. Higuchi Y. Matsukawa T. Murayama D. (2007). Upregulation of IGF-I in the goldfish retinal ganglion cells during the early stage of optic nerve regeneration. Neurochem. Int. 50, 749–756. 10.1016/j.neuint.2007.01.012 17363112

[B21] Kuwahara A. Ozone C. Nakano T. Saito K. Eiraku M. Sasai Y. (2015). Generation of a ciliary margin-like stem cell niche from self-organizing human retinal tissue. Nat. Commun. 6, 6286. 10.1038/ncomms7286 25695148

[B22] Lamba D. A. Karl M. O. Ware C. B. Reh T. A. (2006). Efficient generation of retinal progenitor cells from human embryonic stem cells. Proc. Natl. Acad. Sci. U. S. A. 103, 12769–12774. 10.1073/pnas.0601990103 16908856 PMC1568922

[B23] Lamba D. A. Mcusic A. Hirata R. K. Wang P. R. Russell D. Reh T. A. (2010). Generation, purification and transplantation of photoreceptors derived from human induced pluripotent stem cells. PLoS One 5, e8763. 10.1371/journal.pone.0008763 20098701 PMC2808350

[B24] Lamming D. W. (2016). Inhibition of the mechanistic target of rapamycin (mTOR)-Rapamycin and beyond. Cold Spring Harb. Perspect. Med. 6, a025924. 10.1101/cshperspect.a025924 27048303 PMC4852795

[B25] Langer K. B. Ohlemacher S. K. Phillips M. J. Fligor C. M. Jiang P. Gamm D. M. (2018). Retinal ganglion cell diversity and subtype specification from human pluripotent stem cells. Stem Cell Rep. 10, 1282–1293. 10.1016/j.stemcr.2018.02.010 PMC5998302 29576537

[B26] Lindsley R. C. Gill J. G. Kyba M. Murphy T. L. Murphy K. M. (2006). Canonical Wnt signaling is required for development of embryonic stem cell-derived mesoderm. Development 133, 3787–3796. 10.1242/dev.02551 16943279

[B27] Lingor P. Tönges L. Pieper N. Bermel C. Barski E. Planchamp V. (2008). ROCK inhibition and CNTF interact on intrinsic signalling pathways and differentially regulate survival and regeneration in retinal ganglion cells. Brain 131, 250–263. 10.1093/brain/awm284 18063589

[B28] Liu Y. Wang X. Li W. Zhang Q. Li Y. Zhang Z. (2017). A sensitized IGF1 treatment restores corticospinal axon-dependent functions. Neuron 95, 817–833 e4. 10.1016/j.neuron.2017.07.037 28817801 PMC5582621

[B29] Martinez-Morales J. R. Del Bene F. Nica G. Hammerschmidt M. Bovolenta P. Wittbrodt J. (2005). Differentiation of the vertebrate retina is coordinated by an FGF signaling center. Dev. Cell 8, 565–574. 10.1016/j.devcel.2005.01.022 15809038

[B30] Masterton R. (2013). Sensory integration. Germany: Springer Science & Business Media.

[B31] Mccabe K. L. Gunther E. C. Reh T. A. (1999). The development of the pattern of retinal ganglion cells in the chick retina: Mechanisms that control differentiation. Development 126, 5713–5724. 10.1242/dev.126.24.5713 10572047

[B32] Menu E. Jernberg-Wiklund H. Stromberg T. De Raeve H. Girnita L. Larsson O. (2006). Inhibiting the IGF-1 receptor tyrosine kinase with the cyclolignan PPP: An *in vitro* and *in vivo* study in the 5T33MM mouse model. Blood 107, 655–660. 10.1182/blood-2005-01-0293 16046527

[B33] Meyer J. S. Shearer R. L. Capowski E. E. Wright L. S. Wallace K. A. Mcmillan E. L. (2009). Modeling early retinal development with human embryonic and induced pluripotent stem cells. Proc. Natl. Acad. Sci. U. S. A. 106, 16698–16703. 10.1073/pnas.0905245106 19706890 PMC2757802

[B34] Meyer-Franke A. Kaplan M. R. Pfrieger F. W. Barres B. A. (1995). Characterization of the signaling interactions that promote the survival and growth of developing retinal ganglion cells in culture. Neuron 15, 805–819. 10.1016/0896-6273(95)90172-8 7576630

[B35] Moore D. L. Blackmore M. G. Hu Y. Kaestner K. H. Bixby J. L. Lemmon V. P. (2009). KLF family members regulate intrinsic axon regeneration ability. Science 326, 298–301. 10.1126/science.1175737 19815778 PMC2882032

[B36] Munoz-Sanjuan I. Brivanlou A. H. (2002). Neural induction, the default model and embryonic stem cells. Nat. Rev. Neurosci. 3, 271–280. 10.1038/nrn786 11967557

[B37] Nakano T. Ando S. Takata N. Kawada M. Muguruma K. Sekiguchi K. (2012). Self-formation of optic cups and storable stratified neural retina from human ESCs. Cell Stem Cell 10, 771–785. 10.1016/j.stem.2012.05.009 22704518

[B38] Nelson B. R. Gumuscu B. Hartman B. H. Reh T. A. (2006). Notch activity is downregulated just prior to retinal ganglion cell differentiation. Dev. Neurosci. 28, 128–141. 10.1159/000090759 16508310

[B39] Norsworthy M. W. Bei F. Kawaguchi R. Wang Q. Tran N. M. Li Y. (2017). Sox11 expression promotes regeneration of some retinal ganglion cell types but kills others. Neuron 94, 1112–1120 e4. 10.1016/j.neuron.2017.05.035 28641110 PMC5519288

[B40] Ohlemacher S. K. Sridhar A. Xiao Y. Hochstetler A. E. Sarfarazi M. Cummins T. R. (2016). Stepwise differentiation of retinal ganglion cells from human pluripotent stem cells enables analysis of glaucomatous neurodegeneration. Stem Cells 34, 1553–1562. 10.1002/stem.2356 26996528 PMC4892962

[B41] Osakada F. Jin Z. B. Hirami Y. Ikeda H. Danjyo T. Watanabe K. (2009). *In vitro* differentiation of retinal cells from human pluripotent stem cells by small-molecule induction. J. Cell Sci. 122, 3169–3179. 10.1242/jcs.050393 19671662

[B42] Oswald J. Kegeles E. Minelli T. Volchkov P. Baranov P. (2021). Transplantation of miPSC/mESC-derived retinal ganglion cells into healthy and glaucomatous retinas. Mol. Ther. Methods Clin. Dev. 21, 180–198. 10.1016/j.omtm.2021.03.004 33816648 PMC7994731

[B43] Özdinler P. H. Macklis J. D. (2006). IGF-I specifically enhances axon outgrowth of corticospinal motor neurons. Nat. Neurosci. 9, 1371–1381. 10.1038/nn1789 17057708

[B44] Pankratz M. T. Li X. J. Lavaute T. M. Lyons E. A. Chen X. Zhang S. C. (2007). Directed neural differentiation of human embryonic stem cells via an obligated primitive anterior stage. Stem Cells 25, 1511–1520. 10.1634/stemcells.2006-0707 17332508 PMC2743478

[B45] Parameswaran S. Balasubramanian S. Babai N. Qiu F. Eudy J. D. Thoreson W. B. (2010). Induced pluripotent stem cells generate both retinal ganglion cells and photoreceptors: Therapeutic implications in degenerative changes in glaucoma and age-related macular degeneration. Stem Cells 28, 695–703. 10.1002/stem.320 20166150

[B46] Parameswaran S. Dravid S. M. Teotia P. Krishnamoorthy R. R. Qiu F. Toris C. (2015). Continuous non-cell autonomous reprogramming to generate retinal ganglion cells for glaucomatous neuropathy. Stem Cells 33, 1743–1758. 10.1002/stem.1987 25753398 PMC4524556

[B47] Park K. K. Liu K. Hu Y. Smith P. D. Wang C. Cai B. (2008). Promoting axon regeneration in the adult CNS by modulation of the PTEN/mTOR pathway. Science 322, 963–966. 10.1126/science.1161566 18988856 PMC2652400

[B48] Pera M. F. Andrade J. Houssami S. Reubinoff B. Trounson A. Stanley E. G. (2004). Regulation of human embryonic stem cell differentiation by BMP-2 and its antagonist noggin. J. Cell Sci. 117, 1269–1280. 10.1242/jcs.00970 14996946

[B49] Rapaport D. H. Wong L. L. Wood E. D. Yasumura D. Lavail M. M. (2004). Timing and topography of cell Genesis in the rat retina. J. Comp. Neurol. 474, 304–324. 10.1002/cne.20134 15164429

[B50] Santos-Ferreira T. Llonch S. Borsch O. Postel K. Haas J. Ader M. (2016). Retinal transplantation of photoreceptors results in donor-host cytoplasmic exchange. Nat. Commun. 7, 13028. 10.1038/ncomms13028 27701381 PMC5059459

[B51] Sluch V. M. Chamling X. Liu M. M. Berlinicke C. A. Cheng J. Mitchell K. L. (2017). Enhanced stem cell differentiation and immunopurification of genome engineered human retinal ganglion cells. Stem Cells Transl. Med. 6, 1972–1986. 10.1002/sctm.17-0059 29024560 PMC6430043

[B52] Smith J. R. Vallier L. Lupo G. Alexander M. Harris W. A. Pedersen R. A. (2008). Inhibition of Activin/Nodal signaling promotes specification of human embryonic stem cells into neuroectoderm. Dev. Biol. 313, 107–117. 10.1016/j.ydbio.2007.10.003 18022151

[B53] Sosa L. Dupraz S. Laurino L. Bollati F. Bisbal M. Cáceres A. (2006). IGF-1 receptor is essential for the establishment of hippocampal neuronal polarity. Nat. Neurosci. 9, 993–995. 10.1038/nn1742 16845384

[B54] Spalding K. L. Rush R. A. Harvey A. R. (2004). Target‐derived and locally derived neurotrophins support retinal ganglion cell survival in the neonatal rat retina. J. Neurobiol. 60, 319–327. 10.1002/neu.20028 15281070

[B55] Teotia P. Chopra D. A. Dravid S. M. Van Hook M. J. Qiu F. Morrison J. (2017a). Generation of functional human retinal ganglion cells with target specificity from pluripotent stem cells by chemically defined recapitulation of developmental mechanism. Stem Cells 35, 572–585. 10.1002/stem.2513 27709736 PMC5330843

[B56] Teotia P. Van Hook M. J. Ahmad I. (2017b). A Co-culture Model for Determining the Target Specificity of the de novo Generated Retinal Ganglion Cells. Bio Protoc. 7, e2212. 10.21769/BioProtoc.2212 PMC5484165 28660235

[B57] Teotia P. Van Hook M. J. Fischer D. Ahmad I. (2019). Human retinal ganglion cell axon regeneration by recapitulating developmental mechanisms: Effects of recruitment of the mTOR pathway. Development 146, dev178012. 10.1242/dev.178012 31273087 PMC6633601

[B58] Teotia P. Van Hook M. J. Wichman C. S. Allingham R. R. Hauser M. A. Ahmad I. (2017c). Modeling glaucoma: Retinal ganglion cells generated from induced pluripotent stem cells of patients with SIX6 risk allele show developmental abnormalities. Stem Cells 35, 2239–2252. 10.1002/stem.2675 28792678

[B59] Tran N. M. Shekhar K. Whitney I. E. Jacobi A. Benhar I. Hong G. (2019). Single-cell profiles of retinal ganglion cells differing in resilience to injury reveal neuroprotective genes. Neuron 104, 1039–1055 e12. 10.1016/j.neuron.2019.11.006 31784286 PMC6923571

[B60] Tropepe V. Hitoshi S. Sirard C. Mak T. W. Rossant J. Van Der Kooy D. (2001). Direct neural fate specification from embryonic stem cells: A primitive mammalian neural stem cell stage acquired through a default mechanism. Neuron 30, 65–78. 10.1016/s0896-6273(01)00263-x 11343645

[B61] Tucker B. A. Anfinson K. R. Mullins R. F. Stone E. M. Young M. J. (2013). Use of a synthetic xeno-free culture substrate for induced pluripotent stem cell induction and retinal differentiation. Stem Cells Transl. Med. 2, 16–24. 10.5966/sctm.2012-0040 23283489 PMC3659741

[B62] Vanderwall K. B. Huang K. C. Pan Y. Lavekar S. S. Fligor C. M. Allsop A. R. (2020). Retinal ganglion cells with a glaucoma OPTN(E50K) mutation exhibit neurodegenerative phenotypes when derived from three-dimensional retinal organoids. Stem Cell Rep. 15, 52–66. 10.1016/j.stemcr.2020.05.009 PMC7363877 32531194

[B63] Venugopalan P. Wang Y. Nguyen T. Huang A. Muller K. J. Goldberg J. L. (2016). Transplanted neurons integrate into adult retinas and respond to light. Nat. Commun. 7, 10472. 10.1038/ncomms10472 26843334 PMC4742891

[B64] Volkner M. Zschatzsch M. Rostovskaya M. Overall R. W. Busskamp V. Anastassiadis K. (2016). Retinal organoids from pluripotent stem cells efficiently recapitulate retinogenesis. Stem Cell Rep. 6, 525–538. 10.1016/j.stemcr.2016.03.001 PMC4834051 27050948

[B65] Watanabe K. Ueno M. Kamiya D. Nishiyama A. Matsumura M. Wataya T. (2007). A ROCK inhibitor permits survival of dissociated human embryonic stem cells. Nat. Biotechnol. 25, 681–686. 10.1038/nbt1310 17529971

[B66] Winzeler A. Wang J. T. (2013). Purification and culture of retinal ganglion cells from rodents. Cold Spring Harb. Protoc. 2013, 643–652. 10.1101/pdb.prot074906 23818667

[B67] Xia X. Teotia P. Ahmad I. (2018). Lin28a regulates neurogliogenesis in mammalian retina through the Igf signaling. Dev. Biol. 440, 113–128. 10.1016/j.ydbio.2018.05.007 29758178

[B68] Xiao D. Deng Q. Guo Y. Huang X. Zou M. Zhong J. (2020). Generation of self-organized sensory ganglion organoids and retinal ganglion cells from fibroblasts. Sci. Adv. 6, eaaz5858. eaaz5858. 10.1126/sciadv.aaz5858 32523990 PMC7259937

[B69] Ying Q. L. Nichols J. Chambers I. Smith A. (2003). BMP induction of Id proteins suppresses differentiation and sustains embryonic stem cell self-renewal in collaboration with STAT3. Cell 115, 281–292. 10.1016/s0092-8674(03)00847-x 14636556

[B70] Young R. W. (1985). Cell differentiation in the retina of the mouse. Anat. Rec. 212, 199–205. 10.1002/ar.1092120215 3842042

[B71] Yu P. B. Deng D. Y. Lai C. S. Hong C. C. Cuny G. D. Bouxsein M. L. (2008). BMP type I receptor inhibition reduces heterotopic [corrected] ossification. Nat. Med. 14, 1363–1369. 10.1038/nm.1888 19029982 PMC2846458

[B72] Zhang K. Y. Tuffy C. Mertz J. L. Quillen S. Wechsler L. Quigley H. A. (2021). Role of the internal limiting membrane in structural engraftment and topographic spacing of transplanted human stem cell-derived retinal ganglion cells. Stem Cell Rep. 16, 149–167. 10.1016/j.stemcr.2020.12.001 PMC7897583 33382979

[B73] Zhang X. M. Yang X. J. (2001). Regulation of retinal ganglion cell production by Sonic hedgehog. Development 128, 943–957. 10.1242/dev.128.6.943 11222148 PMC7048390

[B74] Zhang Y. Williams P. R. Jacobi A. Wang C. Goel A. Hirano A. A. (2019). Elevating growth factor responsiveness and axon regeneration by modulating presynaptic inputs. Neuron 103, 39–51 e5. 10.1016/j.neuron.2019.04.033 31122676 PMC7350660

[B75] Zhao X. Liu J. Ahmad I. (2002). Differentiation of embryonic stem cells into retinal neurons. Biochem. Biophys. Res. Commun. 297, 177–184. 10.1016/s0006-291x(02)02126-5 12237099

