# Supplementary material for: Reproducible generation of human retinal ganglion cells from banked retinal progenitor cells: analysis of target recognition and IGF-1-mediated axon regeneration
Source: Front Cell Dev Biol. 2023 Jul 13;11:1214104. doi: 10.3389/fcell.2023.1214104 (PMC10373790; doi:10.3389/fcell.2023.1214104)
Supplement: Supplementary file 1 [file DataSheet2.PDF]

Table S2: List of gene human specific primers for Quantitative Real-Time PCR

Gene	Gene Accession No.	Primer Sequence Forward (5'-3') Reverse (5'-3')	Product Size (bp)
GAPDH	NM_001357943.2	AAGGTGAAGGTCGGAGTCAAC<br>GGGGTCATTGATGGCAACAATA	102
SOX11	NM_003108.4	AGCAAGAAATGCGGCAAGC<br>ATCCAGAAACACGCACTTGAC	180
POU4F2	NM_004575.2	AGCCGGTGAGAATGTGAAAC<br>TGAACACGGGTGATGTCTGT	219
SNCG	NM_001330120.2	ACACCCACCATGGATGTCTT<br>ACAGTGTTGACGCTGCTCAC	239
ISLET1	NM_002202.2	AAACAGGAGCTCCAGCAAAA<br>AGCTACAGGACAGGCCAAGA	157
THY1	NM_006288.4	GAGCAAGAGGAATGGCTCAC<br>TGTGCTGGGCACATTACATT	164
DCC	NM_005215.3	TGCCAGCAGCAGTACATTTC<br>CCATTTCACCCAACGCTAGT	250
ROBO2	NM_001128929.3	CACCCAGAACCCACCATCTAC<br>CACCTGGTTAATTGGCCTCCT	228
NRP1	NM_001244973.2	ACGTGGAAGTCTTCGATGGAG<br>CACCATGTGTTTCGTAGTCAGA	138
EPHB3	NM_004443.4	ATACCAGGTGTGTAATGTGCG<br>CGCTGTCAGCCTCGTAGTAG	188
GAP43	NM_001130064.2	GGGAGGCTTGAGGAAAAATC<br>TCAGCAGCTTGGACATCATC	240
KLF6	NM_001160125.2	CTCCCGAGCCAGAATGATTTT<br>GGCAACAGACCTGCCTAGAG	122
NANOG	NM_001297698.2	TGCTGAGATGCCTCACACGGA<br>TGACCGGGACCTTGTCTTCCTT	155
GATA4	NM_001308093.3	GTGTCCCAGACGTTCTCAGTC<br>GGGAGACGCATAGCCTTGT	102
TBXT	NM_003181.4	TATGAGCCTCGAATCCACATAGT<br>CCTCGTTCTGATAAGCAGTCAC	109
OTX2	NM_001270524.2	CAAAGTGAGACCTGCCAAAAAGA<br>TGGACAAGGGATCTGACAGTG	179
FOXG-1	NM_005249.5	CCGCACCCGTCAATGACTT<br>CCGTCGTAAAACTTGGCAAAG	133
RX	NM_013435.2	GTCCCTAAGCGTGCTTTCAG<br>CATGCCAGGGTCTTGGTACT	54

PAX6	NM_000280.4	TGTGTGCTCTGAAGGTCAGG<br>CTGGAGCTCTGTTTGGAAGG	53
SIX3	NM_005413.4	CCGGAAGAGTTGTCCATGTT<br>CGACTCGTGTTTGTTGATG	171
SIX6	NM_007374.3	CAAGTTCACCAAGGAGTCGC<br>CTGGGGTTAGGGTATGGATCC	237
POU6F2	NM_007252.4	AGTGCCCAGAAGATCAAGCC<br>CATTTCCTGCCCAGAAGGGT	204
RBMPS	NM_006867.4	AGAAGGACGAACAATTGCCCT<br>CACAAGACAGATTGCAGCCG	224
THY1	NM_006288.4	GAGCAAGAGGAATGGCTCAC<br>TGTGCTGGGCACATTACATT	164
SPP1	NM_000582.3	AATCTCCTAGCCCCACAGACC<br>CTTCGGTTGCTGGCAGGT	235
CART	NM_004291.4	GAAAGGGCTCTTTTCCTGCT<br>ATTCTGCCATGCCACATACA	197
OPN4	NM_001030015.3	GCACAACGAAGCAGAGACTCC<br>TGCAGGCTCACAGCATAATC	161
EOMES	NM_001278183.2	AGTGGGTGGATGGGGACAAA<br>CTGAATCCAGCGTCCTTTCC	181
NRL	XM_054376151.1	GGCTCCACACCTTACAGCTC<br>GGCCCATCAACAGGGACTG	212
PROX1	XM_054337737.1	AAAGGACGGTAGGGACAGCAT<br>CCTTGGGGATTCATGGCACTAA	76
