# Supplementary material for: Reproducible generation of human retinal ganglion cells from banked retinal progenitor cells: analysis of target recognition and IGF-1-mediated axon regeneration
Source: Front Cell Dev Biol. 2023 Jul 13;11:1214104. doi: 10.3389/fcell.2023.1214104 (PMC10373790; doi:10.3389/fcell.2023.1214104)
Supplement: Supplementary file 2 [file DataSheet3.PDF]

Table S3: List of gene rat specific primers for Quantitative Real-Time PCR

Gene	Gene Accession No.	Primer Sequence Forward (5'-3') Reverse (5'-3')	Product Size (bp)
Gapdh	NM_017008.4	CCGCATCTTCTTGTGCAGTG<br>ACCAGCTTCCCATTCTCAGC	239
Pou4f2	XM_032884807.1	TTTGAGCGTGGGCCAGTTAG<br>GCTTTTCAAACCTGCAGATGG	206
Pou6f2	NM_001101002.2	ACACTCCCAGCAAGCTCTTC<br>CCTAGTTGTCCCGCCATGTT	226
Islet1	NM_002202.3	CTTTCAGCATTGGCAACCCC<br>GATTGCCGCAACCAACACAT	194
Sncg	XM_039094809.1	CTTCCACACTGGGCTAAGGG<br>CTTGTGCCATGGCTCTCGTA	201
Rbpms	NM_001271244.1	CAGTAGCCCTGAAGTGTGGG<br>GAGCTCCCTCTGTTGGTCTG	229
Thy1	NM_012673.2	CAAGAGGAGGCTGCAAGCTA<br>CAGGCTGAACTCATGCTGGA	217
Sox11	NM_053349.2	ACCCGGACTGGTGCAAGAC<br>CGACTGCTCCATGATCTTCCT	101
Gap43	NM_017195.3	CCCTGTCTCTCCTGCCCTT<br>CCAAAAACTCGCCATAACAACACC	203
Klf6	NM_031642.5	GAGTTCCTCGGTCATTTCCA<br>TGCTTTCAAGTGGGAGCTTT	238
Dcc	XM_039096589.1	ACAACAGGGAGCGAGCTTT<br>GCAGATACAGCGTGCAGGT	205
Robo2	NM_002202.3	GGGCTCAAATAGTCAAGGGCA<br>TGGGATATGTGTGGCTGTGT	195
Cartpt	NM_017110.1	GCGCTATGTTGCAGATCGAA<br>CAGTCACACAGCTTCCCGAT	160
Spp1	NM_012881.2	GCCAGCCAAGGACCAACTAC<br>GCTTCTGAGATGGGTCAGGC	178
Opn4	NM_138860.1	GCCACCTTTCTTTGGTTGGAG<br>ATGATGATGAGCAGGGGGAGGAAG	147
Eomes	XM_039082697.1	GGTATTACCCTGACCCGACC<br>GGGAGTCTCTACCCAGGAGG	182
Nrl	NM_001106036.3	CTGAAAATCTCTCGGGCAACTG<br>TGAGTCCTGATGAGGCTGTGGAAC	132
Prox1	NM_001107201.1	TGACCTGCACTTCCTGTGAG<br>AGAGCAGCCAGCTTTCTCAG	199
