# Supplementary material for: Reproducible generation of human retinal ganglion cells from banked retinal progenitor cells: analysis of target recognition and IGF-1-mediated axon regeneration
Source: Front Cell Dev Biol. 2023 Jul 13;11:1214104. doi: 10.3389/fcell.2023.1214104 (PMC10373790; doi:10.3389/fcell.2023.1214104)
Supplement: Supplementary file 3 [file DataSheet1.PDF]

Table S1: List of Primary and Secondary antibodies used for immunocytochemistry and immunopanning

Name of the protein	Dilution	Catalog No.	Company
PAX6 mouse	1:50	PAX6	DSHB
RX rabbit	1:50	SC-271889	Santa Cruz biotech
TUJ1 mouse	1:500	801202	BioLegend
RFP rabbit	1:500	600-401-379	Rockland
SMI32 mouse	1:500	801701	BioLegend
TAU-1 chicken	1:500	Ab75714	abcam
Brn3b rabbit	1:500	Ab56026	abcam
PSD95 rabbit	1:100	Ab12093	abcam
SNCG rabbit	1:100	Ab55424	abcam
Cholera toxin subunit B Alexa Fluro 488 conjugate	1:500	C34775	Invitrogen
Goat anti-Rabbit Cy3	1:1000	Ab6939	abcam
Goat anti-Rabbit FITC	1:1000	A11008	Invitrogen
Goat anti-Mouse Cy3	1:1000	A10521	Invitrogen
Goat anti-Mouse Alexa Fluro 488	1:1000	A11001	Invitrogen
Goat Anti-Chicken Alexa Fluro 647	1:1000	Ab150171	abcam
Goat anti-mouse IgG	10 µg/ml	115005044	Jacksons laboratory
Goat anti-mouse IgG+IgM	10 µg/ml	115005045	Jacksons laboratory
Macrophage mouse	2 µg/ml	MAB1407P	Millipore
Anti-Thy1.1mouse	2 µg/ml	MABF1961	Millipore
Anti-Thy1.2 mouse	2 µg/ml	MCA02R	Millipore
Anti-HNK-1/NCAM mouse	2 µg/ml	C0678	Sigma
